# Deep Phenotyping and Genetic Characterization of a Cohort of 70 Individuals With 5p Minus Syndrome

**DOI:** 10.3389/fgene.2021.645595

**Published:** 2021-07-30

**Authors:** Julián Nevado, Cristina Bel-Fenellós, Ana Karen Sandoval-Talamantes, Adolfo Hernández, Chantal Biencinto-López, María Luisa Martínez-Fernández, Pilar Barrúz, Fernando Santos-Simarro, María Ángeles Mori-Álvarez, Elena Mansilla, Fé Amalia García-Santiago, Isabel Valcorba, Belén Sáenz-Rico, María Luisa Martínez-Frías, Pablo Lapunzina

**Affiliations:** ^1^CIBERER, Centro de Investigación Biomédica en Red de Enfermedades Raras, Instituto de Salud Carlos (ISCIII), Madrid, Spain; ^2^Instituto de Genética Médica y Molecular (INGEMM)-IdiPAZ, Hospital Universitario La Paz, Madrid, Spain; ^3^ITHACA-European Reference Network-Hospital la Paz, Madrid, Spain; ^4^Departamento de Investigación y Psicología en Educación, Facultad de Educación, Universidad Complutense de Madrid (UCM), Madrid, Spain; ^5^Servicio de Genética, Centro de Rehabilitacion Infantil Teleton (CRIT), Guadalajara, Mexico; ^6^Departamento de Economía Financiera y Actuarial y Estadística, Facultad de Comercio y Turismo, Universidad Complutense de Madrid, Madrid, Spain; ^7^Spanish Collaborative Study of Congenital Malformations (ECEMC), Research Unit on Congenital Anomalies (UIAC), Instituto de Salud Carlos III (ISCIII), Madrid, Spain; ^8^Departamento Estudios Educativos, Facultad de Educación, Universidad Complutense de Madrid, Madrid, Spain

**Keywords:** 5p-minus syndrome, intellectual disabilities, Cri du chat, subtelomeric deletion, behavior problems

## Abstract

Chromosome-5p minus syndrome (5p-Sd, OMIM #123450) formerly known as *Cri du Chat* syndrome results from the loss of genetic material at the distal region of the short arm of chromosome 5. It is a neurodevelopmental disorder of genetic cause. So far, about 400 patients have been reported worldwide. Individuals affected by this syndrome have large phenotypic heterogeneity. However, a specific phenotype has emerged including global developmental delay, microcephaly, delayed speech, some dysmorphic features, and a characteristic and monochromatic high-pitch voice, resembling a cat’s cry. We here describe a cohort of 70 patients with clinical features of 5p- Sd characterized by means of deep phenotyping, SNP arrays, and other genetic approaches. Individuals have a great clinical and molecular heterogeneity, which can be partially explained by the existence of additional significant genomic rearrangements in around 39% of cases. Thus, our data showed significant statistical differences between subpopulations (simple 5p deletions versus 5p deletions plus additional rearrangements) of the cohort. We also determined significant “functional” differences between male and female individuals.

## Introduction

The syndrome of 5p- (5p- Sd) is caused by partial deletion of the short arm of chromosome 5. The size of the deletion is variable ranging from 500 kb or less to 45 Mb (Simmons et al., 1995; Gu et al., 2013; Elmakky et al., 2014). This syndrome is a rare chromosomal disease, with an incidence between 1 in 15,000 and 1 in 50,000 live births (Niebuhr, 1978; Higurashi et al., 1990; Cerruti Mainardi, 2006). The prevalence is higher among females (66%) than males, but the reason is unclear. No differences in prevalence between races or geographical areas have been found or related to prenatal events or age of the parents. In Spain, it is estimated that there are around 500–700 patients (Rodríguez-Caballero et al., 2012, and unpublished data from patient’s Associations). It has been suggested that the great phenotypic variability observed among individuals with this syndrome is related to both the size and the location of the deletion (between 5p15.3 and 5p15.2 bands), since it is a chromosomal region with a large gene content (Nguyen et al., 2015; Correa et al., 2019).

Ninety percent of cases are *de novo*, and 10% are inherited, due to a rearrangement in the parents (unbalanced segregation of translocations, or recombination involving a pericentric inversion, rarely a parental mosaicism, or an inherited terminal deletion). In *de novo* cases, between 80% and 90% are of paternal origin possibly due to chromosome breakage during the formation of male gametes (Cerruti Mainardi et al., 2001). Prenatal diagnoses in 5p- Sd (at 12–16 weeks of gestation) are common because fetuses frequently show abnormal ultrasound signs (∼65–90%) including cerebral abnormalities, cerebellar hypoplasia, absent/hypoplastic nasal bone, hydrops fetalis, ascites or encephalocele, hypospadias, lung dysplasia, IUGR, microcephaly, and micro/retrognatia (Mak et al., 2019; Su et al., 2019; Peng et al., 2020).

Over the past decade, the accuracy of genetic diagnosis and the advances of analytical techniques have allowed to expand the genetic information associated with the short arm of chromosome 5. However, a full map of the involved genes in this syndrome is not completely established, nor the consequences of their haploisufficiency for subjects with 5p- Sd. In this sense, Nguyen et al. (2015) established a role for 11 dose-sensitive genes within the 5p- arm. In five of them, losses may lead to haploinsufficiency (*TERT*, *SEMA5A*, *MARCH6*, *CTNND2*, and *NPR3*), and in the remaining six genes their haploinsufficiency is conditioned by an additional environmental factor (*SLC6A3*, *CDH18*, *CDH12*, *CDH10*, *CDH9*, and *CDH6*). In addition, two additional genes have been suggested to have haplolethal effects (*RICTOR* and *DAB2*).

We here describe the clinical and molecular data of a cohort of 70 unrelated patients with a cytogenetic and/or molecular diagnosis of 5p- Sd. High-resolution single-nucleotide polymorphism (SNP) array, cytogenetic, fluorescence *in situ* hybridization (FISH), and multiplex ligation-probe amplification (MLPA) techniques were applied to most patients, in order to delineate the size, extent, gene content, and additional rearrangements. Genotype–phenotype relationship analyses were also established. A comparison of the clinical features with published patients in the literature and relevant findings that all patients share in this series were also discussed.

## Materials and Methods

### Subjects

**FIGURE 1:**
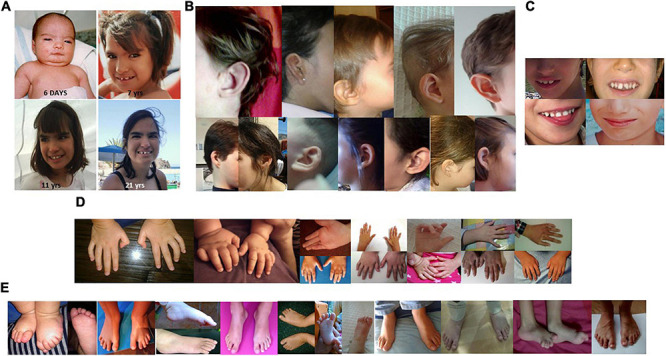
**(A)** Facial features of a patient with 5p minus syndrome at age of 6 days, 7 years, 11 years, and 21 years. **(B)** Details of different ear alterations. **(C)** Details of several dental anomalies, and of a wide mouth. **(D)** Details of some hand and finger anomalies. **(E)** Details of several foot and toe anomalies.

During the period between 2017 and 2020, around 100 patients with 5p minus syndrome, formerly called Cri du chat syndrome (CDCS), were recruited for this study in our center. At this moment, around 30 cases had incomplete either clinical or molecular data and were finally not included in this study. The final cohort is constituted by 70 individuals (see Figure 1 and Supplementary Data). Most of the DNA samples from these patients were extracted and analyzed by SNP arrays at INGEMM (Madrid, Spain), and standard cytogenetic studies were made at the Spanish Collaborative Study of Congenital Malformations Centre (ECEMC) and INGEMM. Clinical information of patients was obtained from the referring physicians by two standardized questionnaires (INGEMM and ECEMC), completed with data of the medical reports, and interviewing most of the parents. Parents or guardians provided informed consent and the Institutional Review Board of our Hospital approved the study (HULP, Madrid, Spain).

### Methods

#### Karyotyping and FISH

Cytogenetic analyses were performed on GTG-banded metaphases at a resolution of about 550 bands according to standard laboratory protocol using Chromosome Kit P (Euroclone, Siziano PV, Italy). FISH was performed according to standard laboratory protocols using commercial subtelomeric 5pter probes, LPU 013SA (covering CTNND2, 5p15.2 and UBE2QL1, 5p15.31, with control at 5q35) and probe FLJ25076 (CytoCell Ltd., Tarrytown, NY, United States) and probe CTNND2 (from Kreatech, Leica, Wetzlar, Germany).

#### Multiplex Ligation-Probe Amplification (MLPA)

We used MLPA Salsa kits P036 and P070 (subtelomeric probes for all chromosomes) and/or P096 and P358 (specific telomeric probes for the 5p arm) to characterize patients with 5p- Sd (MRC Holland, Amsterdam, Netherland). Data analyses were performed according to the protocols supplied by the provider defining relative probe signals by dividing each measured peak area by the sum of all peak areas of the control probes of that sample. The ratio of each peak’s relative probe area was then compared versus a DNA control sample (Promega, United Kingdom), using Coffalyser v.9.4 (MRC Holland).

#### SNP-Array Analysis

**FIGURE 2:**
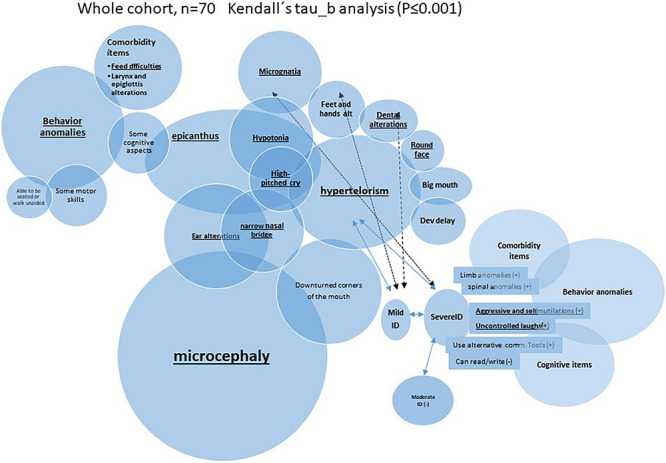
Schematic representation of very significant (*P* ≤ 0.01) inter-correlation among microcephaly and other categoric variables (Kendall’s tau_b analysis was performed).

A genome-wide scan of 850,000 tag SNPs (Infinium CytoSNP-850k BeadChip, Illumina, San Diego, CA, United States) was performed at INGEMM, in the majority of the patients, but three (analyzed by array-CGH at ECEMC). They were analyzed by using the Chromosome Viewer tool contained in the Genome Studio package (Illumina). In Chromosome Viewer, gene call scores <0.15 at any locus were considered “no calls.” In addition, an allele frequency analysis was applied for all SNPs. All genomic positions were established according to the 2009 human genome build 19 (GRCh37/NCBI build 37.1). Deletion sizes were plotted on the genome browser (Figure 2 and Supplementary Data) using the University of California at Santa Cruz Genome Browser^1^.

#### Validation of Global Functional Assessment of the Patients (GFAP)

We estimated individual functional assessment in our cohort by using different features taken from the questionnaires and weighed them by Human Phenotype Ontology (HPO) term frequencies in a numerical scale of five “nuclear” items in the syndrome, based on our clinical experience. A final patient assessment (GFAP) was constructed by the summatory of items “(i) to (v)” as is indicated in Supplementary Table 1, and its validation is explained in the “Results” section.

#### Statistical Analysis

Statistical analysis was performed with SPSS version 25 (IBM Corporation, United States). Descriptive analysis included mean ± SD for continuous variables and frequency tables for categorical variables. These categorical variables were expressed as 1 or 0, indeed grouped as *“ever”* having a given condition compared to *“never”* having the condition, taken from the two questionnaires and curated from medical records. Correlation associations were calculated using Pearson’s linear correlation coefficient (continuous variables) or Spearman’s Rho and Kendall’s tau_b (categorical variables). Comparisons between two groups (as based on sex or to have additional rearrangements) were performed either by Student’s *t*-test (for continuous variables) or by chi-square tests (for categorical ones). For more than two groups, ANOVA analysis (and Bonferroni’s *post hoc* tests) was run for continuous variables, and *z*-tests between column proportions for categorical variables. PCA (principal component analysis) was used to validate our GFAP construct, containing Kaiser–Meyer–Olkin’s measure and Barlett’s test. Ward’s minimum variance method was the criterion used in hierarchical cluster analysis, and the number of clusters was selected using the Bayesian information criterion (BIC) or Akaike information criterion (AIC). A *P*-value (observed significance level) lower than 0.05 or 0.01 was considered to indicate a statistically significant or very significant difference, respectively.

## Results

### Clinical Findings

We evaluate 70 unreported individuals. All but three were from Spain (see Figure 1 and Supplementary Data). The female/male ratio (2.04:1; 47/23) was very similar to previously described cohorts, and ages ranged from birth to 45 years (see Supplementary Table 2). The highest number of individuals with 5p- Sd in our cohort are individuals in the pediatric age (between 0 and 12 years, 77.23%). Descriptive statistics (for continuous variables) and frequencies (for categoric items) are shown in Tables 1, 2, respectively. The mean and median age at evaluation were 8 years and 9 months, and 7 years old, respectively (Table 1).

**TABLE 1:** Descriptive statistics for continuous variables in the whole cohort.

	***N***	**Mean**	**Standard deviation**	**Median**	**Range**
Age at evaluation (years)	70	8.99	8.94	7.00	0.1–45
Gestational age at birth (weeks)	68	38.29	2.59	39.00	30–42
Weight at birth (g)	68	2,602.13	677.55	2,600.00	1,170–4,500
OFC at birth (cm)	68	32.20	2.42	32.00	27–37
Height at birth (cm)	68	45.89	3.90	46.75	32–52
Number of surgeries	70	0.71	1.37	0.0001	0–7
Size of deletion (Mb)	70	20.22	9.29	22.55	0.62–35.01

**N*, number of patients evaluated.*

**TABLE 2:** Frequencies for categorical variables in the whole cohort.

**Categorical variables**	**Female**	**Male**
Gender	47 (67.1%)	23 (32.9%)
	0/“never”/condition not present	1/“ever”/condition present
IUGR	43 (61.4%)	27 (38.6%)
Postnatal growth failure	37 (52.9%)	33 (47.1%)
Microcephaly	11 (15.7%)	59 (84.3%)
Facial asymmetry	61 (87.1%)	99 (12.9%)
Round face	38 (54.3%)	32 (45.7%)
Enlarged face	47 (67.1%)	23 (32.9%)
Hearing problems	40 (57.1%)	30 (42.9%)
Ear alterations	32 (45.7%)	38 (54.3%)
Epicanthus	37 (52.9%)	33 (47.1%)
Ophtalmic anomalies	38 (54.3%)	32 (45.7%)
Prominent superciliary arches	65 (92.9%)	5 (7.1%)
Downslanted palpebral fissures	56 (80.0%)	14 (20.0%)
Hypertelorism	29 (41.4%)	41 (58.6%)
Palpebral fissures size anomalies	64 (91.4%)	6 (8.6%)
Nasal defects	56 (80.0%)	14 (20.0%)
Narrow nasal bridge	26 (37.1%)	44 (62.9%)
Short philtrum	60 (85.7%)	10 (14.3%)
Downturned corners of the mouth	62 (88.6%)	8 (11.4%)
Lip and palate anomalies	63 (90.0%)	7 (10.0%)
Micrognathia	40 (57.1%)	30 (42.9%)
Thick lower lip	54 (77.1%)	16 (22.9%)
Big mouth	52 (74.3%)	18 (25.7%)
Teeth alterations	36 (51.4%)	34 (48.6%)
Neck anomalies	57 (81.4%)	13 (18.6%)
Single palmar crease	58 (82.9%)	12 (17.1%)
Breath problems	43 (61.4%)	27 (38.6%)
Cardiac anomalies	46 (65.7%)	24 (34.3%)
Difficult to feed	42 (60.0%)	28 (40.0%)
Larynx and epiglottis alterations	47 (67.1%)	23 (32.9%)
Gastrointestinal anomalies	31 (44.3%)	39 (55.7%)
Renal anomalies	61 (87.1%)	9 (12.9%)
Genital anomalies	54 (77.1%)	16 (22.9%)
Anal anomalies	65 (92.9%)	5 (7.1%)
Limb anomalies	62 (88.6%)	8 (11.4%)
Alterations in hands or feet	39 (55.7%)	31 (44.3%)
Spinal anomalies	52 (74.3%)	18 (25.7%)
Scoliosis	45 (64.3%)	25 (35.7%)
Joint dislocation includes hip	55 (78.6%)	15 (21.4%)
Joint laxity	39 (55.7%)	31 (44.3%)
Pes cavus	57 (81.4%)	13 (18.6%)
MRI images	18 (25.7%)	52 (74.3%)
Anomalies in MRI images	32 (61.5%)	20 (38.5%)
Hypotonia	21 (30.0%)	49 (70.0%)
Hypertonia	63 (90.0%)	7 (10.0%)
Seizures	66 (94.3%)	4 (5.7%)
Developmental delay	4 (5.7%)	66 (94.3%)
Mild ID	62 (88.6%)	8 (11.4%)
Moderate ID	56 (80.0%)	14 (20.0%)
Severe ID	39 (55.7%)	31 (44.3%)
Behavior anomalies	50 (71.4%)	20 (28.6%)
Autism (ASD)	61 (87.1%)	9 (12.9%)
Hyperactivity	53 (75.7%)	17 (24.3%)
Aggressive and self-mutilation	43 (61.4%)	27 (38.6%)
Stereotypes and repetitive movements	39 (55.7%)	31 (44.3%)
Frustration intolerance	44 (62.9%)	26 (37.1%)
Uncontrolled laughs	50 (71.4%)	20 (28.6%)
Sleeping problems	32 (45.7%)	38 (54.3%)
Cephalic support	19 (27.1%)	51 (72.9%)
Able to stay seated	22 (31.4%)	48 (68.6%)
Able to stay seated unaided	24 (34.3%)	46 (65.7%)
Able to walk unaided	29 (41.4%)	41 (58.6%)
Able to walk with help	23 (32.9%)	47 (67.1%)
Use diapers	38 (55.7%)	31 (44.3%)
Interact with the environment	20 (28.6%)	50 (71.4%)
Can read/write	56 (83.4%)	12 (17.6%)
Use alternative communicative tools	39 (55.7%)	29 (41.4%)
No words at all	44 (65.7%)	26 (34.3%)
Use less than 10 words	43 (63.3%)	25 (36.7%)
Short understandable sentences	52 (76.5%)	16 (23.5%)
High-pitched or horsed cry	31 (44.3%)	39 (55.7%)
Cry w/o sound	68 (97.2%)	2 (2.8%)
Family member no longer work for care	38 (54.3%)	32 (45.7%)
Surgery	46 (67.1%)	24 (32.9%)
Suspicion of pathology prior to diagnosis	48 (68.6%)	22 (31.4%)
Normal electro encephalogram	54 (77.1%)	16 (22.9%)
Normal metabolic screening	53 (75.7%)	17 (24.3%)
Additional duplication	43 (61.4%)	27 (38.6%)

*“1” means *“ever”* having a given condition compared to 0, *“never”* having the condition, taken from either of our two questionnaires, and curated from medical records.*

#### Perinatal and Neonatal Data

Regarding neonatal data, the average gestational age of our cohort was 38.28 ± 2.59 weeks (Table 1). Grossly, 53% (37 subjects) were born between weeks 39th and 40th. Nineteen individuals were born before the 38th week of gestation, three of them below week 32th, and 27 after week 40th. The average birth weight is 2602.13 ± 677.50 g (centile below 5%; Marinescu et al., 2000), which corresponds to the average weight of a neonate of 35th–36th weeks (at centile 50%), and the average length, 45.89 ± 3.90 cm (centile below 5%; Marinescu et al., 2000). Finally, the mean of the cephalic perimeter (OFC) at birth was 32.20 ± 2.42 cm (centile below 5%; Marinescu et al., 2000). More than one-third of subjects were hospitalized at birth. The main causes were prematurity, low weight, and suspected chromosomal abnormality. During the first months of life, several individuals also had feeding difficulties.

#### Postnatal Clinical Findings

The frequencies of clinical features observed in this cohort were recorded using the HPO terms and are listed in Table 3. In Table 3, we also listed data from previous published series of 5p- Sd individuals (Cerruti Mainardi et al., 2006; Van Buggenhout et al., 2000; Rodríguez-Caballero et al., 2012; Espirito Santo et al., 2016; Rodrigues de Medeiros, 2017; Honjo et al., 2018; Chehimi et al., 2020). Figure 1A shows that facial features are not always typical of the syndrome and that a specific *gestalt* is not always present. Nonetheless, microcephaly, large nose bridge, epicanthal folds, hypertelorism, high arched palate, downturned corners of the mouth, round face, ear anomalies (Figure 1B), dental alterations (Figure 1C), short philtrum, micrognathia, and feeding difficulties were present in around or higher than 60% of patients. These should considered, in addition to hypotonia, typical cry/acute voice, breathing problems, and behavior anomalies, as the commonest features in this syndrome (Table 3). On the other hand, alterations of the hands or feet (see Figures 1D,E), hyperlaxity, divergent/convergent strabismus, down-slanting palpebral fissures, stereotypies, gastrointestinal anomalies, short neck, scoliosis, cardiac anomalies, and speech delay were present in 25–59% of the cases and should be considered frequent findings in the syndrome (Table 3).

**TABLE 3:** Phenotypic, comorbidities, and global developmental features of our cohort compared to previous published work.

**Items**	**This study**	**Cerruti Mainardi et al., 2006**	**Espirito Santo et al., 2016**	**Honjo et al., 2018**	**Chehimi et al., 2020**	**Van Buggenhout et al., 2000**	**Rodrigues de Medeiros, 2017**	**Rodríguez-Caballero et al., 2012**	**Total (factored)**
*N*	*N* = 70	*N* = 220	*N* = 6	*N* = 73	*N* = 14	*N* = 7	*N* = 3	*N* = 32	432
Range of age	0.1–45 years	0.8–61 years	6–38 years	9.5–40 years	2–38 years		17–23 years	2–35 years	0.1–45 years
Mean age	8.80 years		16.80 years	13.80 years	13.30 years		19.66 years	14.65 ± 10.19 years	12.32 years
Developmental delay (HP:0001263)	91.40			100.00	100.00		100.00	89.47	95.11
Hypotonia (HP:0003808)	70.00	72.20	100.00				100.00	87.50	99.38
Micrognathia (HP:0000347)	42.90	96.70	100.00		71.00		100.00	90.62	84.26
Epicanthal folds (HP:0000286)	47.10	90.20	83.30			85.71	100.00	93.75	81.67
Large nose bridge (HP:0000446)	62.90	87.20	100.00		57.00	71.42	100.00	93.75	81.78
Typical cry/acute voice (HP:0200046)	55.70	95.90	100.00	94.40	93.00		33.33	93.75	88.25
Hypertelorism (HP:0000316)	58.60	81.40	83.30		71.00	57.14	100.00	93.75	77.16
Aggressive and self- mutilation (HP:0000718)	84.60							65.63	78.65
Behavior anomalies (HP:0012433)	71.40							68.75	70.57
Round face (HP:0000311)	45.70	83.50	100.00		29.00		100.00	25.00	68.62
High arched palate (HP:0000218)	10.00	83.80	100.00		64.00			16/29 (56.17)	65.58
Independent walking	58.60			72.20					65.54
Low-set ears (HP:0000369)	54.30	69.80	33.30			14.28	100.00	78.12	65.20
Microcephaly (HP:0000252)	84.30		66.70		91.00	85.71		6.25	64.93
Use Diapers	44.30			84.00					64.56
Difficult to feed (HP:0011968)	40.00		60.00	80.30				71.87	62.55
Downturned corners of the mouth (HP:0002714)	11.40						81.00		64.20
Dental anomalies (HP:0000164)	48.60	75.00					100.00	13/23 (56.52)	58.30
Short philtrum (HP:0000322)	14.30	60.50			86.00			96.87	55.40
Hyperlaxity (HP:0002761)	44.30							78.12	54.91
Downslanting palpebrals fissures (HP:0200005)	20.00	56.90	83.30						48.71
Strabismus divergent/convergent (HP:0000486)	45.70	47.50	100.00			42.86		17/31 (54.83)	48.67
Short neck (HP:0000470)	18.60	56.20	33.30				100.00		47.38
Stereotypies (HP:0000733) (HP:0008762)	44.30			40.30					42.25
No words at all (HP:0001344)	35.30			47.20					41.37
Hyperactivity (HP:0000752)	24.30							71.87	39.22
Scoliosis (HP:0002944)	*35.70*		33.30	28.80			*42.60*		38.41
Breath problems (HP:0002098)	*38.60*						100.00		38.38
Cardiac anomalies (HP:0115080)	*34.30*	35.80		31.50			100.00	14/29 (48.27)	36.23
Gastro-intestinal anomalies (HP:0011024)	*55.70*						*21.40*	65.62	33.25
Facial asymmetry (HP:0000324)	12.90					57.14		68.75	31.28
Larynx and epiglottis alterations (HP:0001600)	32.90							28.00	31.36
Enlarged face (HP:0100729)	32.90							13.79	26.90
Alterations of the hands or feets (HP:0011297) (HP:0002813)	44.30		83.30				19.50		26.66
Short understandable sentences	28.50			18.10					22.74
Single palmar crease (HP:0000954)	17.10						100.00		20.51
Autism (HP:0000717)	12.90							25.80	19.95
Genital anomalies (HP:0000078)	22.90			8.20					15.40
Hypertonia (HP:0001276)	*10.00*							15.62	11.76
Renal anomalies (HP:0004742)	*12.90*			5.50					12.67

*Data are expressed in percentages. All columns are weighed based on the number of total cases in each item. *N*, is number of patient evaluated.*

It is remarkable that many of those called “nuclear clinical features” (the most frequent findings) seemed to be interrelated among them. Indeed, it showed significant positive correlation among them when a Kendall’s tau_b analysis was performed. For example, microcephaly presented in more than 65% of the cases correlated with epicanthus, narrow nasal bridge, or ear alterations. As an example, Figure 2 summarizes some of those very significant intra-correlations (*P* ≤ 0.01), e.g., microcephaly. The expandend analyses of these correlations are summarized in Supplementary Table 3.

Brain MRI studies were performed in almost 75% of the individuals, though only 28.6% showed some kind of alterations (Table 2), including cerebellar amygdala herniation, abnormalities of the corpus callosum (ranging from thinness to agenesis), frontal horn ectasia, brainstem hypoplasia, dilated ventricular system, cysts, or hydrocephalus. Electroencephalograms showed normal results in only a reduced number of individuals (12/70, 22.90%) (Table 2).

Speech abilities (evaluated only in patients aged ≥3 years; *n* = 56) showed severe abnormalities in the majority of patients (40/56; ∼71.50%). In fact, 30.35% of patients (17/56) had no speech at all, 41.07% (23/56) had an elementary vocabulary of 10 words or less, and 28.57% (16/56) were reported to have a mild vocabulary and the ability to use limited phrases for a short and comprehensible conversation (Tables 2, 3).

As examples of comorbidities, almost 33% of the cohort undertook at least one surgery (ranging from one to seven, Table 1) and include ventricular septal defect (VSD), percutaneous closure of the patent ductus arteriosus, closure of open foramen oval, duodenal atresia, strabismus, and inguinal hernia (the most frequent).

### Genetic Findings

#### Breakpoint Data Analysis

SNP-array analysis was performed in most cases except three patients who had comparative genomic hybridization (CGH array). Genomic coordinates for microdeletions affecting the short arm of chromosome 5 and other genomic rearrangements are listed in Table 4. A graphic representation of the deletions is shown in Supplementary Figure 2. Briefly, the average size of the losses was 20.21 ± 9.28 Mb (range 0.62–35.01 Mb). SNP arrays established the existence of other clinically significant genomic rearrangements in almost 39% of the patients (Table 4); most of them were not previously detected by cytogenetic studies (see Table 4 and Supplementary Data). Most subjects had terminal deletions (65/70, 92.85%), and five individuals carried interstitial deletions (represented in Supplementary Figure 2B). Among the terminal deletions, 16 of them (22.85%), had an additional terminal duplication in other chromosome, which could result from a possible translocation (*de novo* or inherited). Cytogenetic analysis of the parents allowed us to establish whether the rearrangements were familiar (6 cases) or *de novo* (10 cases). In one case, the 5p deletion was inherited from a maternal mosaicism (6.5% of cells in blood) unknown until the moment of diagnosis of the child. We found patients with additional terminal deletions in other chromosomes (two cases, 2.85%) and additional rearrangements at chromosome-5 nearby the deletions (seven cases, 10%). Finally, three children inherited from their mothers a simple, isolated terminal deletion.

**TABLE 4:** Genomic coordinates from all the rearrangements (GRCh37, hg19).

	**Deletions**				**Duplications**			
**Individual**	**Chromosome**	**Coordinates start**	**Coordinates end**	**Size (Mb)**	**Chromosome**	**Coordinates start**	**Coordinates end**	**Size (Mb)**
1	5p15.33-p15.1	25328	17981563	17.98	Xp22.33	169805	2123990	2.12
2	5p15.33-p14.3	25328	22446214	22.44	10q25.3-q26.3	115402474	135434319	20.08
3	5p15.33-p15.2	25328	12995938	12.99				
4	5p15.33-p14.1	25328	28779357	28.77				
5	5p15.33-p14.3	25328	22956970	22.95				
6	5p15.33-p15.31	25328	9438756	9.43	Xq28	154933691	155236712	3.72
7	5p15.33-p13.2	25328	34602269	34.60				
	5p13.2**	34602654	36816661	2.21				
8	5p15.33-p13.3	25328	31853346	31.85				
9	5p15.33-p13.2	25328	35015297	35.02				
10	5p15.33-p14.1	25328	25290077	25.30				
11*	5p15.32-p15.1	4928318	15418957	10.49				
12	5p15.33-p14.2	25328	24430251	24.43				
13	5p15.33-p14.1	25328	28783716	28.78				
14	5p15.33-p15.32	25328	4938756	4.94				
15	5p15.33-p13.3	25328	34986724	34.98				
16	5p15.33-p15.1	25328	17665529	17.60	12p11.21	32875287	33056330	0.18
17	5p15.33-p14.1	25328	25027051	25.02				
18	5p15.33-p15.1	25328	15913112	15.91	8p23.3p-23.1	176617	11860710	11.86
19	5p15.33-p14.1	25328	25396006	25.40	5p14.1	25409917	28435493	3.025
20	5p15.33-p15.1	25328	15808138	15.81				
21	5p15.33-p15.2	25328	12978580	12.98	10q-26.11-q26.3	121556072	135425341	13.87
22	5p15.33-p15.2	25328	11037420	11.037				
23	5p15.33-p15-32	25328	4356789	4.35	5p15.33-p15.32	4355708	4969019	0.60
					5p15.31	6325532	6642356	0.30
24	5p15.33-p14.1	25328	27108052	27.10				
25	5p15.33-p14.3	25328	21872896	21.88	9p24.3-p22.1	46587	19713500	19.7
26	5p15.33-p14.3	25328	22658970	22.65	8p23.2-p11.23	2061877	34908297	34.94
27	5p15.33-p15.2	25328	14360436	14.36				
28	5p15.33-p13.3	25328	29485091	29.48				
29	5p15.33-p13.3	25328	29292854	29.29	1p13.1-p12	117594464	117989275	0.38
30	5p15.33-p13.3	25328	32130401	32.13				
31	5p15.33-p14.1	25328	27708038	27.71	18p11.32	13034	2656248	2.65
32	5p15.33-p14.1	25328	28147535	28.15				
33	5p15.33-p14.1	25328	26622073	26.62	5p13.3-p13.2	26695268	34019038	7.67
34	5p15.33-p14.1	25328	28796749	28.79				
35*	5p15.33-p15.1	560000	17509888	16.95				
36	5p15.33-p14.1	25328	25821865	25.82				
37	5p15.33-p14.3	25328	21504581	21.50	8p23.3-p23.2	164984	752709	0.75
					22q11.21	25661725	25914593	0.25
38	5p15.33-p15.32	25328	4610206	4.61	5q35.1	169708691	169893751	0.18
					5q35.1-q35.3	171656863	180693344	9.03
39	5p15.33-p15.1	25328	15922302	15.92				
40	5p15.33-p14.2	25328	24438467	24.43				
41	5p15.33-p14.1	25328	25135494	25.13	11q22.1	100578089	100870339	0.29
42	5p15.33-p15.1	25328	15022112	15.02	9p24.3-p21.3	162931	23232287	23.24
43	5p15.33-p14.1	25328	28464893	28.46	18p11.32-p11.31	141896	6785383	6.78
44	5p15.33-p14.2	25328	24247673	24.24				
45	5p15.33-p15.1	25328	17704161	17.70	5p14.3	19970119	20370847	0.4
46	5p15.33-p13.2	25328	28929082	28.92	5p13.3-p13.2	28968268	34497445	5.53
47	5p15.33-p15.1	25328	16410000	16.41				
48	5p15.33-p13.3	25328	31131409	31.13				
49	5p15.33-p15.2	25328	14270500	14.27				
50	5p15.33-p15.2	25328	14061184	14.06	7p22.3-p11.3	44935-	11857504	11.86
51	5p15.33-p15.1	25328	16661282	16.67				
52	5p15.33	25328	618586	0.62	2q36.3-2q27.3	22990625	243048760	13.14
	2q36.3	228782976-	229669531	0.89				
53	5p15.33-p15.31	25328	7125022	7.12				
54	5p15.33-p15.31	25328	7125022	7.12				
55	5p15.33-p13.3	25328	30210500	30.21				
56	5p15.33-p15.32	25328	4610206	4.61	2p15.3-p25.2	14238	4698068	4.68
57*	5p15.1-p13.3	17509888	32677299	15.17				
58	5p15.33-p13.3	25328	34119847	34.11	Xp22.31	6447911-	8135053	
59	5p15.33-p13.3	25328	30480030	30.48				
60	NA	NA	NA	NA				
61	5p15.33-p15.1	25328	15652433	15.65	11p15.5-p15.4	75328-	10525251	10.5
62	NA	NA	NA	NA				
63	5p15.33-p15.13.3	25328	30445734	30.44				
64	5p15.33-p15.32	25328	4610206	4.61	2p15.3-p25.2	14238	4698068	4.68
65	5p15.33-p15.32	25328	4610206	4.61	2p15.3-p25.2	14238	4698068	4.68
66*	5p142-p13.2	23383424-	36609355	13.22				
67	5p15.33-p15.31	25328	7125022	7.12				
68**	5p15.33-p13.3	25328	29292854	29.29				
69	5p15.33-p15.32	25328	5014883	5.01				
70*	5p15.2-p13.3	9860050	33760050	23.09				

*Means, interstitial cases. **Means, mosaics.

### “Functional” Findings

#### Individual GFAP

The great heterogeneity observed in patients with this syndrome together with the high number of other significant genomic rearrangements (besides the 5p deletions) raised the question whether the presence of these additional rearrangements may modulate functionally the clinical features in this syndrome and to explain the high intra-cohort variability. We proposed a graduation of the individual global assessment of functionality (GFAP), constructing one continuous variable, based on the frequency of the different “nuclear” clinical items (i to v, see section “Materials and Methods”), and our clinical experience in the syndrome.

**FIGURE 3:**
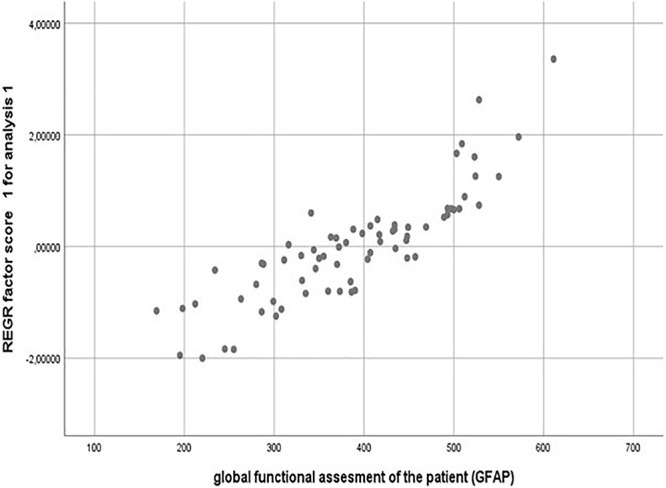
Validation of a GFAP construct by PCA (principal component analysis) statistical approach. Pearson correlation value = 0.846; *P* = 0.001.

To verify this GFAP scale construction, a statistical combined analysis of Kaiser–Meyer–Olkin’s, Bartlett’s, and principal component analysis (PCA) test were performed to detect the best way of association between these grouped clinical features. Indeed, the first principal component (PCA 1) from PCA weighed the major score of the variance, supporting that PCA1 can be written as a weighted average of the five original variables. Finally, Pearson correlation analysis showed that PCA1 and the item GFAP are very significantly correlated (Pearson correlation value = 0.846; *P* = 0.001). The dispersion plot shows the strong linear correlation among them and therefore justifies GFAP as a valid construct (Figure 3).

Table 5 shows the median and mean ± SD values for GFAP and its intermediates “functional” components for the whole cohort, and both subpopulations: simple deletions (47 cases) and patients with deletions and additional rearrangements (mainly duplications, 23 cases).

**TABLE 5:** Mean (±SD) and median of GFAP (Global Functional Assessment of the Patient) and its intermediates (items “i” to “v”) from the whole cohort and subpopulations of 5p- individuals.

	**Whole cohort**		**Single 5p deletions**		**5p deletions plus additional rearrangements**	
**“Functional” variable**	**Median**	**Mean ± *SD***	**Median**	**Mean ± *SD***	**Median**	**Mean ± *SD***
GFAP	387.60	388.40 ± 100.00	361.50	362.89 ± 98.50	404.00	398.70 ± 92.00
(i) Developmental delay items corrected by age	251.00	244.60 ± 66.40	228.50	233.80 ± 59.50	249.00	229.10 ± 73.00
(ii) Behavioral alteration items	7.50	13.40 ± 15.40	7.00	10.80 ± 12.10	7.00	15.00 ± 19.60
(iii) Dysmorphic items	24.00	21.10 ± 11.60	24.00	20.30 ± 11.80	24.00	19.00 ± 0.60
(iv) Communication skills	48.00	54.10 ± 25.80	45.00	50.50 ± 25.80	60.00	58.20 ± 23.80
(v) Comorbidity items	47.00	55.20 ± 47.00	47.00	48.80 ± 38.80	67.00	61.50 ± 37.50

*Values are expressed in arbitrary units. Higher values are associated with a worse functional prognosis.*

### Comparative Analysis

#### Among Subpopulations With and Without Additional Rearrangements

A chi-square test was performed to compare categoric variables in both groups: simple (isolated) 5p deletions and those including 5p deletions and additional rearrangements (mainly duplications). Interestingly, the presence of additional rearrangements may exert significant differences on prenatal and postnatal growth delay findings, cardiac anomalies, and *speech abilities* in the expressive language (Table 6, *P* ≤ 0.05, at CI 95%). Remarkably, other findings became significant at CI of 90%, such as cleft lip/palate, renal anomalies, autistic spectrum disorders (ASD), or breathing difficulties (Table 6).

**TABLE 6:** Comparison between subpopulations in 5p- individuals, regarding categorical variables taken.

**Chi-square test**					
	**Simple 5p dels *N* = 47**	**5p dels + addt rearrangement *N* = 23**	**Value**	**df**	**Sig. asymptotic (bilateral)**
IUGR	14	13	1.701	1	0.019*
Postnatal growth failure	16	17	3.716	1	0.036*
Cardiac anomalies	10	14	6.020	1	0.014*
Short understandable sentences	14	2	5.299	1	0.021*
Cleft lip-palate anomalies	2	5	3.543	1	0.060^$^
Renal anomalies	3	6	3.441	1	0.064^$^
ASD	3	6	3.441	1	0.064^$^
Prominent superciliary arches	5	0	3.381	1	0.066^$^
Breath problems	13	14	3.272	1	0.070^$^
No words at all	10	11	3.187	1	0.074^$^
Interact with the environment	34	16	3.189	1	0.074^$^

* means significant *p*-value ≤ 0.05; ^$^ means possible tendency, significant at CI 90% (data not shown). *N* is the number of patients evaluated.

**FIGURE 4:**
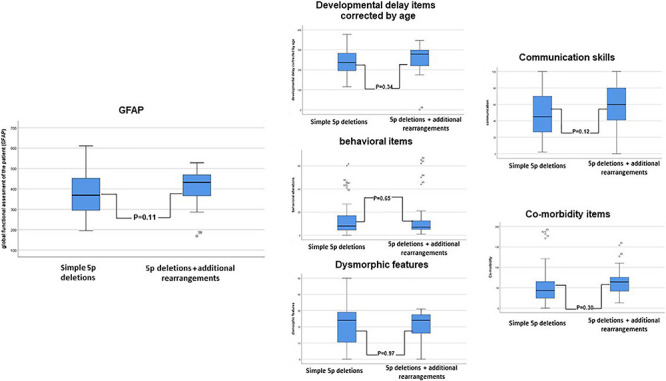
Schematic representation of the comparison between subpopulations in 5p- individuals (5p deletions vs. 5p deletion plus additional rearrangements) in the “functional” constructed GFAP (Global functional Assessment of the Patient) and its intermediates (developmental delay items corrected by age, behavioral items, dysmorphic items, communication skills, and comorbidity items). A Student *t*-test was performed.

Ward cluster analysis allowed us to compare the frequencies of these variables in both subpopulations. We denote that better figures (low percentages) were more represented in the simple 5p deletion group, but with motor items, slightly better than in the group with additional rearrangements (Table 5 and Supplementary Data). Although the simple deletion group had a higher size of 5p deletions on average (see Supplementary Table 5), no statistical significant differences could be observed between the two different subpopulations (*Student t-test*, see Figure 4).

**FIGURE 5:**
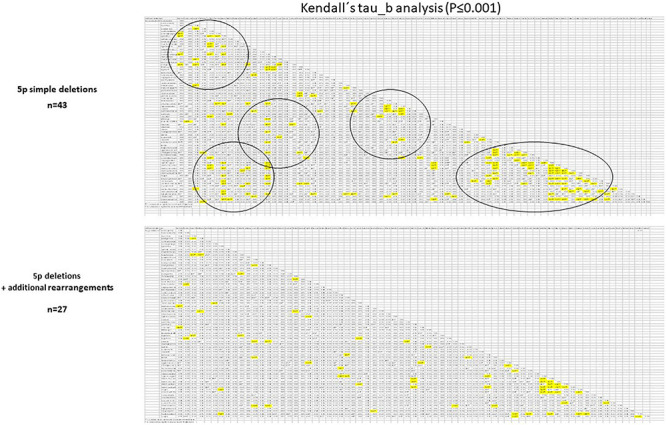
Schematic representation of the comparison between subpopulations in 5p- individuals (5p deletions vs. 5p deletion plus additional rearrangements) in categoric variables using Kendall’s tau_b statistical analysis. Very significant differences (*P* ≤ 0.01) were denoted in bold. Circles denote significant differences among variables observed in the 5p deletion group and absent in the 5p deletion + additional rearrangement group.

We also performed an association analysis among categoric variables in both subpopulations by Kendall’s tau_b analysis (expanded analysis for the whole cohort and subgroups is presented in Supplementary Table 3). Interestingly, some of the observed correlations in the simple 5p deletion group disappeared in the group with additional rearrangements (Figure 5). A more specific example for three of these categoric variables is presented in Supplementary Figure 3.

#### Genotype–Phenotype Correlations

**FIGURE 6:**
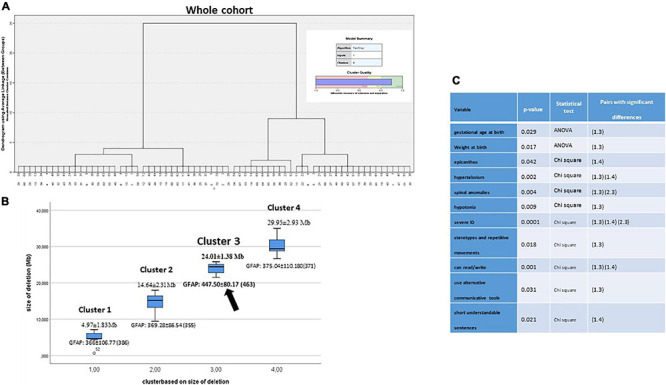
**(A)** Ward’s hierarchical cluster of the whole cohort by size of the deletions. BIC and AIC determined to be grouped by four clusters. **(B)** Plot segregation ordered by deletion size in Mb. Every cluster showed the GFAP value. **(C)** ANOVA analysis for continuous variables or by chi-square test for categoric variables was performed to establish the existence of significant differences between clusters. Further, Bonferroni’s test and *z*-test for previous significant variables revealed cluster pairs with significant differences among them.

We made Ward’s hierarchical cluster analysis using the item “size of deletion” as unique variable, in order to verify how individuals (initially, from the whole cohort) group according to their deletion size. At the end, individuals were grouped in four clusters (the number was established by BIC and AIC algorithms), as follows: 4.97 ± 1.83 Mb, 14.64 ± 2.31 Mb, 24.01 ± 1.38 Mb, and 29.95 ± 2.93 Mb (Figures 6A,B). ANOVA analysis discarded a significant correlation between the size of the deletion and the functional item, GFAP, or any of its intermediates (*P* = 0.07 at CI 95%, data not shown). However, ANOVA analysis for continuous variables or by chi-square test for categoric variables shows the existence of significant differences between clusters in a few variables, mostly related to perinatal parameters, some dysmorphic features, behavior, and cognitive features (Figure 6C). Further, Bonferroni’s and *z*-tests for previous significant variables revealed that cluster 3 (size 24.01 ± 1.38 Mb; 5p15.1–p14.1) is the most represented among the cluster pairs with significant differences among them (Figure 6C).

**FIGURE 7:**
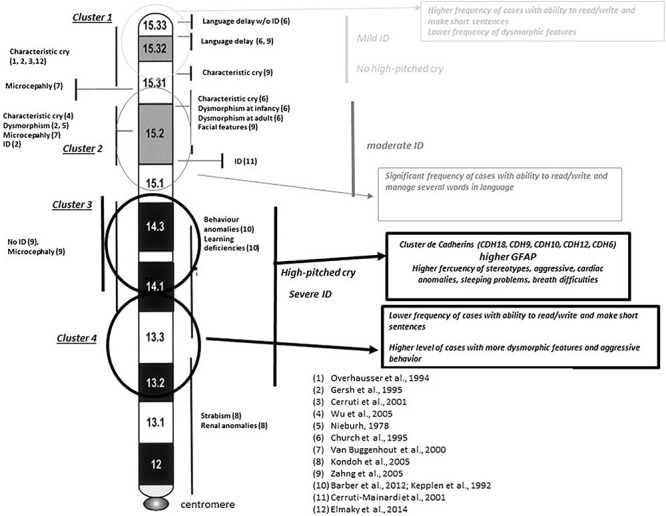
Integrative map for clusters shown in Figure 6. Comparisons with previously reported critical regions for phenotype sings at 5p minus syndrome (references refer to clinical symptoms reported in individual families with interstitial deletions in different reported works). Circles represent the areas mapped for the different clusters obtained in Ward’s analysis. Major findings observed in our study are in italic text. Chromosome bands are reported according to ISCN. ID, intellectual disability.

When Ward’s clusters were dissected by item frequencies (in percentages), higher percentages (normally associated with a worse prognostic) seemed to be mapped, in cluster 3 too (Table 6 and Supplementary Data). However, expressive language (followed by item, *the ability to make short sentences*) or the ability to write or read was associated preferentially with clusters 1 and 2 (11/16 individuals, 69%). Figure 7 shows how the four clusters integrate into suggested functional areas of chromosome-5p of several previously published data. We observed some relevant mapping findings such as the item speech delay, which was mapped at the beginning of the telomere.

We further analyzed these possible differences among clusters (by size of deletion) in the two subpopulations of 5p- Sd individuals (simple, isolated 5p deletions vs. 5p deletions plus additional rearrangements), using the same statistical approach presented above. Supplementary Figure 4A showed a similar result for simple deletions. We also found intra-cluster significant differences for some variables, with cluster 3 again as the most representative cluster for significant differences in the pair of cluster comparisons (Figure 4A and Supplementary Data). Remarkably, one of these variables that showed differences among clusters was GFAP. For analysis of the group with additional rearrangements, we generated only two clusters for comparison (due to the number of individuals) but also denoted significant differences between clusters “A” and “B” (now, cluster “A” aggregates clusters 1 and 2 and “B” clusters 3 and 4, Supplementary Figure 4B).

Finally, Pearson correlation analysis established that the size of the deletion inversely correlated with some neonatal parameters, such as weight or OFC (*P* ≤ 0.001), and almost with birth length (*P* = 0.061). However, the most significant genotype/phenotype correlation was observed between size of the deletions and gender (males, 15.79 ± 8.79 vs. females, 22.38 ± 8.84. Student *t*-test, *P* = 0.004).

#### Male vs. Female Comparative Analysis

A chi-square test was performed for the whole cohort and two of the subpopulations. Table 7i shows the statistic significant differences between males and females in the whole cohort. These differences were mostly related to growth delay (prenatal and postnatal), dysmorphic features, some spinal comorbidities, and behavioral and cognitive aspects. In addition, Ward cluster analysis between males and females showed the worst frequencies (in percentages) in females (Table 7 and Supplementary Data). As we expected, neonatal data at birth showed also significant differences among gender and weight and OFC (*P* ≤ 0.01 and *P* ≤ 0.05, respectively, Student *t*-test) or with length at birth (*P* = 0.074, Student *t*-test). Most remarkably, there were also significant differences at the functional GFAP (*P* = 0.05, Student *t*-test). These differences showed higher values of frequencies (mainly, a worse prognosis) in females. Similarly, we compared male vs. female significant differences for all categoric variables (in both isolated deletions and deletions + additional rearrangements) (Tables 7ii,iii). Significant correlations were found among gender, independently of the group. The only significant difference in common was intrauterine growth retardation (IUGR). Again, the most remarkable finding with significant differences in the simple 5p deletion group was GFAP (Table 7ii), but not in the group with additional rearrangements (Table 7iii). On the other hand, patients with additional rearrangements also showed significant differences in neonatal data, such as weight or OFC at birth, again as the whole cohort, showing better numbers in males than in females. Expanded Student *t*-test analysis is shown in Supplementary Table 8.

**TABLE 7:** Comparison between male and female 5p- individuals, regarding categorical and continuous variables taken by means of the chi-square and Student *t*-test, respectively, in **(i)** the whole cohort, **(ii)** simple 5p deletions, and **(iii)** 5p deletions plus additional rearrangements.

**(i) The whole cohort**					

**Chi-square**					

	**Male**	**Female**	**Value**	**df**	**Sig. asymptotic (bilateral)**
IUGR	3	27	9.42	1	0.002**
Postnatal growth failure	7	26	3.838	1	0.050*
Round face	6	26	5.318	1	0.021*
Enlarged face	12	11	5.794	1	0.016*
Neck anomalies	1	12	4.583	1	0.032*
Alterations of the fingers or toes	6	25	4.598	1	0.032*
Spinal anomalies	2	16	5.194	1	0.023*
Scoliosis	4	21	5.009	1	0.035*
Severe ID	5	26	7.058	1	0.008**
Aggressive and self-mutilation	4	23	6.486	1	0.011*
Sleeping problems	8	30	5.255	1	0.022*
Can read/write	7	5	3.911	1	0.048*
Short understandable sentences	9	7	4.701	1	0.030*
Mild ID	5	3	3.598	1	0.058^$^

**Student *t***					

	**Male**		**Female**		**Sig.**

Weight at birth (g)	2925.91	689.24	2447.28	621.33	0.006**
OFC at birth (cm)	33.17	2.27	31.74	2.37	0.021*
GFAP	358.87	73.39	402.94	108.60	0.050*
Height at birth (cm)	47.11	3.79	45.31	3.86	0.074^$^

**(ii) Simple 5p deletions**					

**Chi-square**					

	**Male**	**Female**	**Value**	**df**	**Sig. asymptotic (bilateral)**

IUGR	2	12	4.669	1	0.0031**
Faillure to thrive	3	13	3.716	1	0.054
Larynx and epiglottis alterations	1	10	5.002	1	0.025*
Severe ID	3	14	4.605	1	0.032*
Aggressive and self-mutilation	3	14	4.605	1	0.032*
Sleeping problems	4	16	4.740	1	0.029*
Spinal anomalies	1	8	3.318	1	0.069^$^

**Student *t***					

	**Male**		**Female**		**Sig.**

GFAP	336.31	61.36	396.37	123.10	0.040*
Behavioral item, as component of GFAP	7.73	11.72	22.63	13.77	0.063^$^

**(iii) 5p deletions + additional rearrangements**					

**Chi-square**					

	**Male**	**Female**	**Value**	**df**	**Sig. asymptotic (bilateral)**

IUGR	1	12	4.34	1	0.037*
Round face	1	12	1.73	1	0.037*
Enlarged face	6	4	9.60	1	0.002**

**(iii) 5p deletions + additional rearrangements**					

**Chi-square**					

	**Male**	**Female**	**Value**	**df**	**Sig. asymptotic (bilateral)**

Ophthalmological anomalies	1	12	4.34	1	0.037*
Short understandable sentences	2	0	5.59	1	0.018*
Auditive problems	5	6	3.68	1	0.055^$^

**Student *t***					

	**Male**		**Female**		**Sig.**

Weight at birth (g)	3314.29	686.24	2469.60	586.85	0.004**
OFC at birth (cm)	34.79	1.55	31.44	2.11	0.001**
Size of the deletions	10.25	6.81	21.60	8.69	0.004**

* means significant *p*-value ≤ 0.05. ** means significant *p*-value ≤ 0.01. ^$^ means possible tendency, significant at CI 90% (data not shown).

## Discussion

In this work, we describe the largest cohort of Spanish patients with 5p- Sd and one of the largest series of these patients so far, characterized by means of CMA and other genetic approaches, such as cytogenetics, MLPA, and FISH. Although its prevalence is still unknown, it was estimated around 1:15,000–50,000 (Niebuhr, 1978; Higurashi et al., 1990; Cerruti Mainardi et al., 2006). Our data showed that 5p- individuals may have a high clinical variability that is accompanied also by a high genetic heterogeneity. In fact, individuals with 5p- syndrome do not always carry a single rearrangement. In our cohort, around 39% of the individuals presented an additional clinically significant genomic rearrangement, mainly a duplication in other chromosomes. In other cases, additional deletions and duplications can be observed nearby the main 5p deletion (seven cases), probably as a result of a complex rearrangement, as it has been previously suggested (Gu et al., 2013). These additional rearrangements raised the question of whether additional genomic rearrangements may have a role in the syndrome, and thus, it may explain part of its variability, or if individuals with additional rearrangements should be considered as having 5p minus syndrome.

We described and compared our cohort with other previously reported series in terms of clinical features. Some limitations of this study come from information taken from the questionnaires filled up by parents or caregivers. This could explain part of these differences among subjects. We strongly recommend systematic codification of clinical features using the HPO system.

We think that frequency-weighed HPO terms grouped in five main nuclear features of the syndrome will help clinicians to describe 5p- Sd patients (Table 3). We built a quotation scale called GFAP (see sections “Materials and Methods” and “Results”). We compared this “functional” GFAP and its intermediate components in order to establish putative significant differences between both subpopulations: simple, isolated 5p deletions and 5p deletions with additional rearrangements. However, no statistical significant differences (*Student t-test*) could be observed between the two different subpopulations for the GFAP variable, although several significant differences could be denoted among other clinical features. The most relevant were cardiac anomalies and speech delay and the presence of additional rearrangements. Regarding behavioral aspects, there were significant differences among subpopulations in sleeping problems, stereotypical or aggressive behavior, and number of behavioral problems, being more common in the group with an additional genomic rearrangement. Thus, the latter showed better numbers in some cognitive items than simple 5p deletions. Altogether and based on statistic analysis, the presence of additional duplications did not have any significant representation over the whole phenotype of the 5p- patient, but it might have specific contributions for some clinical findings such as growth delay (either prenatal or postnatal) as well as cardiac anomalies.

### Genotype–Phenotype Correlations

Some authors have previously stated that the severity of the phenotype and the cognitive delay of 5p- Sd were associated with an increased size of the deletion at chromosome 5p (Wilkins et al., 1983; Cornish et al., 1999; Cerruti Mainardi et al., 2001). However, this fact was not confirmed by others (Marinescu et al., 1999; Espirito Santo et al., 2016). Thus, this aspect is still controversial. We used our “functional” construct GFAP to validate this hypothesis. Our data supported these genotype–phenotype correlations only in simple deletions. Since there is a scant number of publications in this syndrome incorporating microarray data, it cannot be discussed whether this fact has occurred in other cohorts. For instance, Cerruti Mainardi et al. (2006) analyzed genotype–phenotype correlations but only in patients with isolated 5p terminal deletions (151/185 cases).

Furthermore, if a part of the huge phenotypic variability observed among 5p- individuals was not related to the size of the deletion, the other possibility may be established by the location of the deletion, since it is a chromosomal region with an important gene content. Our data supported that specific regions at chromosome 5p may have more significant roles in the syndrome than others. Our analysis of clusters (by size of the deletion) showed that cluster 3 was the most relevant among the cluster pairs with statistically significant differences, both in the whole cohort and subpopulation groups. In fact, the worst frequencies of most categorical items, as well as GFAP and its intermediates in cluster 3, seem to support this observation. Cluster 3 mapped at 18–25 Mb from the telomere (chromosomal bands 5p15.1–5p14.1). Among the genes mapping in this area were the cadherin (CDH) cluster, including *CDH10*, *CDH9*, *CDH12*, *CDH18*, and *CDH6*, strongly associated with this syndrome. This CDH cluster has been described to be conditionally haploinsufficient and depend on other genetic or environmental factors leading to an abnormal phenotype. This is an interesting fact and could also explain part of the variability observed in 5p-Sd. Other genes in this region are *FBXL7*, *MARCH11*, *FAM134B*, *MYO10*, *DROSHA*, *PDZD2*, *GOLPH3*, *MTMR12*, *ZFR*, *SUB1*, *NPR3*, and *TARS*. All genes have a significant level of haploinsufficiency (see Supplementary Table 9). However, we cannot rule out a role for other genes such as *CTNND2*, *TERT*, and *MED10*, commonly deleted in 5p- Sd and associated with neuronal development/function and cellular death. The smallest region of overlap patients with interstitial deletions pointed out to two potential regions, one mapping at these genes and the other in the cadherin cluster. Additional interstitial cases and functional assays are needed to unreveal the role of all these genes.

Speech skills (evaluated only in patients aged >3 years) yielded that the potentially affected region is near to the telomere (5p15.33–5p15.31) supporting previous findings (Church et al., 1995; Zhang et al., 2005). High frequencies for most of the behavioral findings seemed to be associated with clusters 3 and 4 in our Ward’s cluster analysis and supported previous studies for its hypothetical mapping (Barber et al., 2011). On the other hand, our data also showed some discrepancies with previous studies. In our study, high-pitched cry seemed to map at p14.3–p13.2 bands versus bands p15.33–p15.31 (Overhauser et al., 1994; Gersh et al., 1995; Cerruti Mainardi et al., 2001), p15.31 (Zhang et al., 2005), p15.31–p15.2 (Church et al., 1995), or p15.2 (Wu et al., 2005) in other previous reports.

### Gender as a Differentiating Factor: Correlations Depending on Gender

A suspicion of putative cognitive and “functional” differences between males and females patients has been constantly suggested to us by parents, caregivers, and several clinical specialists. This is the first report showing “functional” differences between males and females in 5p- Sd individuals. We found that some of the clinical features analyzed showed statistically significant differences among males and females, for instance in the GFAP variable. Thus, we denoted worse functional scores and higher deletion sizes in females than in males using Ward’s cluster analysis. Additional efforts with systematic cognitive–behavioral evaluations of the patients must be performed in order to assign more precise differences.

The reason why the ratio female–male is 2:1 is still unknown. One of the most relevant differences between genders is the mean value for size of the deletions. Interestingly, Ward’s cluster analysis allowed us to observe how the female/male ratio was modulated by the different sizes of the deletions in the clusters (Tables 5, 7 and Supplementary Data). The number of males in these clusters decreased drastically when the size of the deletion increased over 15 Mb. This fact may suggest a different, possibly lethal, effect of deletions over 15 Mb in males and might explain the differences among the female/male ratio in this syndrome. In fact, miscarriages are frequent in this syndrome. This is not an unusual effect because other genes at 5p13.1, such as *RICTOR* and *DAB2*, have been suggested to be haplolethals (Peng et al., 2020) and may explain how deletions do not expand in size, more than 39 Mb from the telomere. However, we cannot rule out any other additional genetic or epigenetic effect in males, affecting chromosomal bands 5p15.1–p13.2. In fact, an aberrant DNA methylation in Cri du chat syndrome related to development conditions has been already suggested (Naumonova et al., 2018).

## Conclusion

Summing up, we here report a large series of patients with 5p minus syndrome emphasizing some phenotype–genotype correlations. Remarkably, we found statistically significant “functional” differences among males and females. We also dissected subpopulations in 5p- Sd based on the presence/absence of clinical significant additional rearrangements, besides losses at the 5p arm. The presence of these additional rearrangements may have a role modulating part of the phenotype in the syndrome.

Finally, we recommend combining typical karyotyping with CMA as the definitive method for a precise diagnosis of 5p- Sd, in order to provide a more accurate genetic counseling for these families.

## Data Availability Statement

The original contributions presented in the study are publicly available. This data can be found in the DECIPHER database (https://www.deciphergenomics.org/) with the following accession numbers: 436269 to 436336 corresponding to cases 5pIMG01-5pIMG70.

## Ethics Statement

The studies involving human participants were reviewed and approved by Institutional Review Board Hospital la Paz. Written informed consent to participate in this study was provided by the participants’ legal guardian/next of kin. Written informed consent was obtained from the individual(s) for the publication of any potentially identifiable images or data included in this article.

## Author Contributions

JN conceived the presented idea, completed the data analysis, and wrote the manuscript. JN and PL designed the study. JN coordinated the data acquisition and collected the data from the second questionnaires. AS-T created and managed the Final 5p- database. PL, AH, CB-L, and BS-R assisted with the data management and statistics. CB-L analyzed the conductual and cognitive profiles of the patients. JN, PB, and MM-Á managed the SNP and CGH microarrays at the INGEMM. EM, IV, and FG-S provided the FISH and karyotyping studies at INGEMM. FS-S and PL provided and explored several patients at INGEMM. MM-Fr and MM-Fe created the first questionnaire and managed some of the patients’ cytogenetic analysis. All authors contributed to the article and approved the submitted version.

## Conflict of Interest

The authors declare that the research was conducted in the absence of any commercial or financial relationships that could be construed as a potential conflict of interest.

## Publisher’s Note

All claims expressed in this article are solely those of the authors and do not necessarily represent those of their affiliated organizations, or those of the publisher, the editors and the reviewers. Any product that may be evaluated in this article, or claim that may be made by its manufacturer, is not guaranteed or endorsed by the publisher.

## Footnotes

1 http://genome.ucsc.edu/; Kent et al., 2002.
