# Supplementary material for: Deep Phenotyping and Genetic Characterization of a Cohort of 70 Individuals With 5p Minus Syndrome
Source: Front Genet. 2021 Jul 30;12:645595. doi: 10.3389/fgene.2021.645595 (PMC8362798; doi:10.3389/fgene.2021.645595)
Supplement: Supplementary file 1 [file Table_1.DOCX]

**Table 1 Supplemental data.** Construction of GFAP (Global Functional Assessment of the Patient). Variables were taken from any of two questionnaires, and values were annotated from our clinic experience.

| **VARIABLES** | **to have** | **not to have** |
| --- | --- | --- |
| **i) Affecting Developemental Delay corrected by age:** |  |  |
| *IUGR* | 20 points | 0 points |
| *postnatal growth failure* | 20 points | 0 points |
| *gestational week under the mean value* | 5-20 points | 0 points |
| *weight at birth under the mean value* | 5-20 points | 0 points |
| *height at birth under the mean value* | 5-20 points | 0 points |
| *OFC at birth under the mean value* | 5-20 points | 0 points |
| *Microcephaly* | 20 points | 0 points |
| *Feed difficulties* | 20 points | 0 points |
| *suction problems* | 20 points | 0 points |
| *gastroesophagic reflux* | 20 points | 0 points |
| *anomalies in limbs* | 20 points | 0 points |
| *altrations in fingers and toes* | 20 points | 0 points |
| *espinal anomalies* | 20 points | 0 points |
| *scoliosis* | 20 points | 0 points |
| *MRI anomalies* | 20 points | 0 points |
| *hypotonia* | 20 points | 0 points |
| *hypertonia* | 20 points | 0 points |
| *seizures* | 20 points | 0 points |
| *developmental delay* | 20 points | 0 points |
| *Intellectual disability (ID):* |  |  |
| *light ID* | 5 points | 0 points |
| *moderate ID* | 10points | 0 points |
| *severe ID* | 20 points | 0 points |
| *sleep problems* | 20 points | 0 points |
| *cephalic support* | 20 points | 0 points |
| *able to be seated with help* | 0 points | 20 points |
| *able to be seated unaided* | 0 points | 20 points |
| *able to walk with help* | 0 points | 20 points |
| *able to walk unaided* | 0 points | 20 points |
| *use diapers* | 20 points | 0 points |
| *interact to environment* | 0 points | 20 points |
|  |  |  |
| **ii) Behavioural problems:** |  |  |
| *MRI anomailies* | 20 points | 0 points |
| *hypotonia* | 20 points | 0 points |
| *hypertonia* | 20 points | 0 points |
| *seizures* | 20 points | 0 points |
| *conductual problems* | 1 points | 0 points |
| *autism* | 1 points | 0 points |
| *hyperactivity* | 1 points | 0 points |
| *aggressive* | 1 points | 0 points |
| *stereotypes* | 1 points | 0 points |
| *frustration intolerance* | 1 points | 0 points |
| *uncontrollled laughs* | 1 points | 0 points |
| *high-pitched cry* | 1 points | 0 points |
| *no sound cry* | 1 points | 0 points |
|  |  |  |
| **iii) dismorphic items:** |  |  |
| *epicanthus* | 5 points | 0 points |
| *hypertelorism* | 5 points | 0 points |
| *Narrow nasal bridge* | 5 points | 0 points |
| *Any other one* | 1 points | 0 points |
|  |  |  |
| **iv) Communication items** |  |  |
| *able to write or read* | 0 points | 20 points |
| *no words at all* | 20 points | 0 points |
| *use alternative communication tools* | 20 points | 0 points |
| *use less tha 10 words* | 20 points | 0 points |
| *use short comprenhensible sentences* | 0 points | 20 points |
| *use less tha 50 words* | 5 points | 0 points |
| *a few words* | 5 points | 0 points |
| *fluid language* | 0 points | 10 points |
|  |  |  |
| **v) Comorbidity** |  |  |
| *Microcephaly* | 20 points | 0 points |
| *Auditive problems* | 1 points | 0 points |
| *optalmologic alterations* | 1 points | 0 points |
| *recurrent respiratory infections* | 1 points | 0 points |
| *vascular anomalies* | 1 points | 0 points |
| *cardiac problems* | 1 points | 0 points |
| *feed problems* | 20 points | 0 points |
| *suction problems* | 20 points | 0 points |
| *gastroesophagic reflux* | 20 points | 0 points |
| *bowel atresia* | 1 points | 0 points |
| *Esophagic atresia* | 1 points | 0 points |
| *larynx alterations* | 1 points | 0 points |
| epiglottis alterations | 1 points | 0 points |
| *umbilical herniae* | 1 points | 0 points |
| *gastrointestinal problems* | 1 points | 0 points |
| *renal anomalies* | 1 points | 0 points |
| *inguinal herniae* | 1 points | 0 points |
| *genitalia anomalies* | 1 points | 0 points |
| *anal anomalies* | 1 points | 0 points |
| *spinal anomalies* | 20 points | 0 points |
| *scoliosis* | 20 points | 0 points |
| *hyperlaxity* | 1 points | 0 points |
| *joint dislocation* | 20 points | 0 points |
| *skin problems* | 1 points | 0 points |
| *MRI anomalies* | 20 points | 0 points |
| *hypotonia* | 20 points | 0 points |
| *hypertonia* | 20 points | 0 points |
| *seizures* | 20 points | 0 points |
| *Intellectual disability* | 20 points | 0 points |
| *sleeping problems* | 20 points | 0 points |
| *normal EEG* | 0 points | 20 points |
| *normal Electro cardiogram* | 0 points | 20 points |
| *normal metabolic screening* | 0 points | 20 points |
