# Supplementary material for: Deep Phenotyping and Genetic Characterization of a Cohort of 70 Individuals With 5p Minus Syndrome
Source: Front Genet. 2021 Jul 30;12:645595. doi: 10.3389/fgene.2021.645595 (PMC8362798; doi:10.3389/fgene.2021.645595)
Supplement: Supplementary file 2 [file Table_2.DOCX]

**Tabla 2. Supplemental data --** Total sample distribution by age and gender

| Age | Male | Female |  | Age at evaluation (years) | N | % |
| --- | --- | --- | --- | --- | --- | --- |
| 0 | **1** | **5** |  |  |  |  |
| 1 | **2** | **4** |  | **0-2** | **15** | **21,51** |
| 2 | **1** | **2** |  |  |  |  |
| 3 | **1** | **2** |  |  |  |  |
| 4 | **1** | **3** |  | **2.1-4** | **7** | **10** |
| 5 | **3** | **2** |  |  |  |  |
| 6 | **1** | **3** |  |  |  |  |
| 7 | **1** | **4** |  |  |  |  |
| 8 | **3** | **1** |  |  |  |  |
| 9 | **1** | **4** |  | **4.1-12** | **32** | **45,72** |
| 10 | **1** | **2** |  |  |  |  |
| 11 | **1** | **2** |  |  |  |  |
| 12 | **1** | **2** |  |  |  |  |
| 13 | **3** | **4** |  |  |  |  |
| 14 | **1** | **1** |  | **12.1-18** | **11** | **17,15** |
| 16 | **0** | **2** |  |  |  |  |
| 20 | **0** | **1** |  |  |  |  |
| 35 | **1** | **0** |  |  |  |  |
| 38 | **0** | **1** |  | **+ 18** | **5** | **5,71** |
| 40 | **0** | **1** |  |  |  |  |
| 45 | **0** | **1** |  |  |  |  |
| TOTAL | **23** | **47** |  |  | **70** | **100** |
