# Supplementary material for: Deep Phenotyping and Genetic Characterization of a Cohort of 70 Individuals With 5p Minus Syndrome
Source: Front Genet. 2021 Jul 30;12:645595. doi: 10.3389/fgene.2021.645595 (PMC8362798; doi:10.3389/fgene.2021.645595)
Supplement: Supplementary file 4 [file Table_4.DOCX]

**Table 3. Supplemental Data.** Cytogenetic, FISH and MLPA data in the whole cohort

| Sujeto | Cariotipo/FISH | Banda | Otros estudios |
| --- | --- | --- | --- |
| 1 | 46,XY, del(5)(p15.2).ish del(5)(p15.2)(CTNND2),del(5)(p15.31) (FLJ25076-), del(5)(pter) | 5p15.1-pter |  |
| 2 | 46,XX.ish der(5)t(5;10)(p15q26)mat(C84C11-, D10S2490+) | 5p14-ter | mlpa 5psubtel (P070B1,P036E1)x1; mlpa 10qsubtel (P070B1,P036E1)x3 |
| 3 | 46,XY,(del)(5)(:p15.1->ter) | 5p15.2-pter |  |
| 4 | 46,XX,(del)(5) | 5p14-ter |  |
| 5 | 46,XX,(del)(5)(:p15.2) | 5p15.2-ter |  |
| 6 | 46,XX,(del)(5)(p15) |  |  |
| 7 | 46,XX,(del)(5)(p13 | 5p15.2-ter |  |
| 8 | 46,XX, Normal |  |  |
| 9 | 46,XX,del (5) (p13) |  |  |
| 10 | NA |  |  |
| 11 | NA |  |  |
| 12 | 46,XY,(del)(5)(p15.1p15.3).ish del(5)(p15q26)(D5S721/D5S23-) | 5p15.1-pter |  |
| 13 | 46,XX,del (5) (p13->pter) |  |  |
| 14 | NA |  |  |
| 15 | NA |  |  |
| 16 | 46,XX,(del)(5)(p14) | 5p14-ter |  |
| 17 | 46,XX,(del)(5)(p14pter). ish del (p14pter (tel5p-) |  | mlpa 5psubtel (P070B1,P036E1)x1 |
| 18 | 46,XY,(del)(5)(p15.2).ish del(5) (p15.2p15.2p15.31) (CTNND2-) (FLJ25076-) | 5p15.2-ter |  |
| 19 | 46,XX,(del)(5)(p13) | 5p13-ter |  |
| 20 | NA |  |  |
| 21 | 46, XY normal | 5p15.3-ter | mlpa P245(5p)x1 |
| 22 | 46,XY,(del)(5)(p15.2).ish del(5)(p15.2)(CDCR-) | 5p15.2-ter |  |
| 23 | 46, XX, der t(5;16) ¿ (p15.3; p11.2) r (16)? (p112q24) dn, ish. der t(5;16) (C84C11/t3- , 5pter)(D5S2807+, 5qter) | 5p15.3-ter |  |
| 24 | 46,XX,(del)(5)(p13) | 5p13-ter |  |
| 25 | 46,XX, del(5)(p15.2).ish del(5)(p15.2)(CTNND2-), del(5) (p15.31)(FLJ25076-),del(5)(pter-) | 5p15.2-ter |  |
| 26 | 46,XX.ish der(5)t(5;8)(p15.1;p21.1)pat | 5p15.1-ter | mlpa 5psubtel (P070B1,P036E1)x1; mlpa 8psubtel (P070B1,P036E1)x3 |
| 27 | 46,XX,(del)(5)(p15). Ish, del (5) (p15.2) (D5S721-, D5S23-) | 5p13.3-ter |  |
| 28 | 46,XY,(del)(5)(13.3) | 5p13.3-ter |  |
| 29 | 46,XX,(del)(5p)(13).ish (tel5p-) 5p12.2 (D5S23x2) | 5p13-ter |  |
| 30 | 46,XX.ish del (5) (p15.1;p21.1) (816F10 +; 921F7 -) | 5p14-ter |  |
| 31 | 46,XX,(del)(5p13-ter) | 5p13-ter |  |
| 32 | 46,XX.ish 5p15.2(CDCR).ish tel(5p-) dn | 5p15.2-ter |  |
| 33 | NA |  |  |
| 34 | NA |  |  |
| 35 | NA |  |  |
| 36 | 46,XX,(del)(5)(p21.3pter) | 5p13.2-ter |  |
| 37 | 46, XX, Normal (AF) and 46,XX,(del)(5)(p15.1pter) (blood 1 yrs) |  |  |
| 38 | 46, XX, normal and 46,XX,add(5)(p15.3).ish der(5)del(5)(p15.3)(C84C11/T3-) dup(5)(q35.3)(G53508/T7++) | 5p15.3-pter |  |
| 39 | 46,XX, del(5)(p15.2) | 5p15.2-ter |  |
| 40 | NA |  |  |
| 41 | 46,XX,(del)(5)(p14-pter) | 5p14-ter |  |
| 42 | 46,XY, del(5)(p15.1)?, ish del(5)(p15.2)(CTNND2-) (5q31 (EGR1)x2) | 5p15.2-ter |  |
| 43 | 46,XX,(del)(5)(p14) | 5p14-ter |  |
| 44 | NA |  |  |
| 45 | NA |  |  |
| 46 | 46XX del(5) p14 |  |  |
| 47 | 46,XX, del(5)(p15.2) |  |  |
| 48 | 46,XX, del(5) |  |  |
| 49 | 46,XY, del(5)(p15.1) |  | mlpa P096 (5p)x1 |
| 50 | NA |  | mlpa P070,P036 (5p;7p)x1, x3; mlpa P096 (5p)x1 |
| 51 | 46,XX, del(5)(pter>p15.1), ish. del (5p) (pter>p15.1) (D5S117-) | 5p15.33- | mlpa P096 (5p)x1 |
| 52 | 46, XY der t(2;5) (q37.1;p15.3), ish. drl (5p) t(2;5) (q37.1;p15.3) (wcp5+, CTNND+, D5S2488-, D2S2142+) (pter>p15.1) (D5S117-) |  | mlpa P096(5p)x1; mlpa P036 (5p)x1;(2q)x3 |
| 53 | 46,XY, del(5)(p15.31) mat |  | mlpa 5psubtel (P070B1,P036E1)x1; mlpa (P358)x1 |
| 54 | 46, XY normal |  | mlpa 5psubtel (P070B1,P036E1)x1; mlpa (P358)x1 |
| 55 | 46,XY, del (5)(p13.2?)[25] / 46, XY [25] | 5p15.33-p15.2 | mlpa P096(5p)x1; |
| 56 | 46, XX normal, ish. der t(2;5) (p25.3;p15.3) (D2S2147+;D5S2488-) |  | mlpa (P208)x3; mlpa P036 (5p)x1 |
| 57 | 46,XX, del(5)(p14p15.1), ish. del (5) (p15.2) (D5S117+) |  | mlpa (P245)x2; mlpa (P036)x2 |
| 58 | 46,XX,(del)(5)(p13) | 5p15.3-ter |  |
| 59 | 46,XX,(del)(5)(p13), ish. del (5) (p15.2p15.31) (CTNND2-, FLJ25076-) | 5p15.33-p13.3 |  |
| 60 | 46,XY, del (5) |  |  |
| 61 | 46, XX normal.ish. der(5) t(5;11) (p15.3;p15.5) pat ()D5S2488-,D11S1363+) |  |  |
| 62 | 46,XX, del (5) |  |  |
| 63 | 46,XX,(del)(5)(p13) |  |  |
| 64 | 46, XY normal, ish. der t(2;5) (p25.3;p15.3) (D2S2147+;D5S2488-) |  | mlpa (P208)x3; mlpa P096 (5p)x1 |
| 65 | 46, XY normal, ish. der t(2;5) (p25.3;p15.3) (D2S2147+;D5S2488-) |  | mlpa (P208)x3; mlpa P096 (5p)x1 |
| 66 | 46,XY, del(5) (p13.2p14.2) |  | mlpa 5psubtel (P070B1,P036E1)x1; mlpa (P358)x1 |
| 67 | 46,XX, del(5)(p15.31?), ish. Del (5) (p15.33) (C834c11T7-) |  | mlpa 5psubtel (P070B1,P036E1)x1; mlpa (P358)x1 |
| 68 | 46, XX, normal, ish. del (5) (p15.3) (D5S2488-) [7] /46, XX [93] |  |  |
| 69 | 46, XY, normal |  |  |
| 70 | 46,XX, del(5) |  |  |
