# Supplementary material for: Deep Phenotyping and Genetic Characterization of a Cohort of 70 Individuals With 5p Minus Syndrome
Source: Front Genet. 2021 Jul 30;12:645595. doi: 10.3389/fgene.2021.645595 (PMC8362798; doi:10.3389/fgene.2021.645595)
Supplement: Supplementary file 5 [file Table_5.DOCX]

**Table 5. Supplemental data.** *Ward´s Cluster analysis in simple 5p deletions versus 5p deletion+ rearrangements.*

| **Items** | simple 5p deletions | 5p deletion plus addtl. rearrangements |
| --- | --- | --- |
| **gender** | 31F/16M  (ratio, 1.94:1) | 16F/07M  (ratio, 2.28:1) |
| **size of deletion (Mb)** | 29.1±8.72 (24.71) range 5.01 -35.01 | 16.69±9.70 (15.91)  range 0.62 -34.11 |
| **Prenatal/Neonatal** |  |  |
| **IUGR** | 14/47 (29.78%) | 11/23 (47.82%)* |
| **postnatal growth failure** | 15/47 (31.91%) | 10/23 (43.47%)* |
| **gestational week** | 38.08±2.56(39) | 38.65±2.69(39) |
| **weight at birth** | 2518.93±679(2600) | 2689±696(2670) |
| **height at birth** | 46.17±3.65(47) | 45.50±4.35(46) |
| **OFC at birth** | 32.01±2.46(32) | 32.55±2.38(33) |
| **Postnatal** |  |  |
| **hypotonia** | 27/47 (57.44%) | 12/23 (52.17%) |
| **hypertonia** | 4/47 (8.51%) | 5/23 (21.73%) |
| **developmental delay** | 22/47 (46.08%) | 14/23 (60.86%) |
| **light ID** | 5/47 (10.64%) | 2/23 (8.69%) |
| **moderate ID** | 9/47 (19.15%) | 3/23 (13.04%) |
| **severe ID** | 16/47 (34.04%) | 7/23 (30.43%) |
| **GFAP** | 362.89±98.59(361.5) | 398.72±92.00(404) |
| ***Developmental items*** | 233.82±59.50(228.5) | 229.172±73.00(249) |
| ***Behavioral alt.*** | 10.80±12.10(7) | 15.00±19.61(7) |
| ***Dysmorphic feat.*** | 20.34±11.80(24) | 19.04±10.64(24) |
| ***Communication*** | 50.50±25.80(45) | 58.21±23.82(60) |
| ***Comorbidity*** | 48.80±38.80(44.5) | 61.52±37.56(67) |
| **Co-morbidity features** |  |  |
| **MRI anomalies** | 13/34 (38.23%) | 7/18 (38.89%) |
| **seizures** | 1/47 (2.13%) | 3/23 (13.04%) |
| **high-pitched cry** | 22/47(40%) | 13/23(50%) |
| **cry w/o sound** | 2/47(4.26%) | 0/23 (0%) |
| **breathing difficulties** | 12/47 (25.53%) | 10/23 (43.47%) |
| **cardiac anomalies** | 15/47 (25.53%) | 10/23 (43.47%)* |
| **difficult to feed** | 12/47 (31.91%) | 12/23 (52.15%) |
| **Laringyx and epiglottis alt.** | 11/47 (23.41%) | 11/23 (47.82%) |
| **gastrointest. alt.** | 22/47(40.00%) | 12/23 (52.15%) |
| **Renal anomalies** | 4/47 (8.51%) | 5/23 (21.73%) |
| **hyperlaxity** | 17/47 (25.53%) | 16/23 (69.16%) |
| **auditive problems** | 19/47 (29.79%) | 13/23(56.52%) |
| **ophtalmological prob.** | 17/47 (25.53%) | 8/23 (34.81%) |
| **genitalia anomalies** | 12/47 (27.66%) | 7/23 (30.43%) |
| **sleeping problems** | 12/47 (27.66%) | 8/23 (34.81%) |
| **SOCIAL** |  |  |
| **a family member quit job** | 24/47 (55.22%) | 8/23 (34.81%) |
| **COGNITIVE** |  |  |
| **use diapers** | 18/47 (38.29%) | 12/23 (52.15%) |
| **int. with environment** | 35/47 (74.46%) | 14/23 (60.86%) |
| **can read/write** | 10/45 (22.22%) | 3/22 (13.63%) |
| **use alternative comm. tools** | 17/47 (25.53%) | 10/21 (47.61%) |
| **no words** | 13/47 (27.66%) | 7/21 (33.33%) |
| **less than 10 words** | 17/47 (25.53%) | 7/21 (33.33%) |
| **make short sentences** | 14/47 (29.78%)* | 2/21 (2.52%) |
| **MOTOR** |  |  |
| **cephalic sosten** | 36/47 (76.58%) | 15/23 (62.11%) |
| **stay seated with help** | 33/47 (70.21%) | 13/23(56.52%) |
| **stay seated unaided** | 33/47 (70.21%) | 13/23(56.52%) |
| **walk unaided** | 28/47 (59.57%) | 12/23 (52.15%) |
| **walK with help** | 30/47 (63.83%) | 14/23 (60.86%) |
| **DYSMORPHIC FEAT.** |  |  |
| **Microcephaly** | 35/47 (73.90%) | 16/23 (63.37%) |
| **facial assimetry** | 6/46 (13.04%) | 3/23 (13.04%) |
| **round face** | 18/46 (39.13%) | 5/23 (21.73%) |
| **enlarged face** | 7/46 (15.17%) | 5/23 (21.73%) |
| **ear malformations** | 24/47 (51.06%) | 11/23 (47.82%) |
| **epicanthus** | 19/47 (29.79%) | 6/23 (26.08%) |
| **hypertelorism** | 23/47 (48.9%) | 10/23 (43.47%) |
| **narrow nasal bridge** | 22/47 (46.8%) | 9/23 (39.13%) |
| **short philtrum** | 3/47 (6.38%) | 4/23 (17.41%) |
| **cleft lip/palate, ojival** | 1/47 (2.13%) | 6/23 (26.08%) |
| **micrognathia** | 36/47 (38.24%) | 9/23 (39.13%) |
| **big mouth** | 06/47 (13.83%) | 6/23 (26.08%) |
| **neck anomalies** | 07/47 (14.89%) | 3/23 (13.04%) |
| **teeth anomalies** | 22/47(40.00%) | 10/23 (43.47%) |
| **downslanted palpebral fisures** | 8/47 (17.02%) | 3/23 (13.04%) |
| **BEHAVIOUR ALT.** | 31/47 (65.96%) | 16/23 (63.37%) |
| **ASD** | 3/47 (6.38%) | 5/23 (21.73%) |
| **hyperactivity** | 10/47 (21.27%) | 6/23 (26.08%) |
| **aggressive** | 16/47 (34.04%) | 7/23 (30.43%) |
| **stereotypes** | 19/47 (29.79%) | 9/23 (39.13%) |
| **Frustration intolerance** | 11/47 (23.41%) | 7/23 (30.43%) |
| **uncontrolled laughs** | 12/47 (27.66%) | 7/23 (30.43%) |

* means significant differences among subpopulations (Chi square test)
