# Supplementary material for: Deep Phenotyping and Genetic Characterization of a Cohort of 70 Individuals With 5p Minus Syndrome
Source: Front Genet. 2021 Jul 30;12:645595. doi: 10.3389/fgene.2021.645595 (PMC8362798; doi:10.3389/fgene.2021.645595)
Supplement: Supplementary file 6 [file Table_6.DOCX]

**Table.6. Supplemental Data.** *Ward ´s Clusters analysis (by size of the deletion).*

| **Variable** | **Cluster 1** | **Cluster 2** | **Cluster 3** | **Cluster 4** |
| --- | --- | --- | --- | --- |
| **gender** | 5F/6M | 11F/10M | 12F/4M | 19F/3M |
| **size of deletion (Mb)** | 4.97±1.83 (4.61) range 0.62 -7.13 | 14.64±2.31(15.02) range 9.43 -17.98 | 24.01±1.38(24.43) range 21.50 -25.82 | 29.95±2.93(29.29)range 26.54 -35.01 |
| **additional duplication** | 5/11 (45.45%) | 9/21 (42.85%) | 6/16(37.50%) | 7/22 (31.81%) |
| **Prenatal/Neonatal** |  |  |  |  |
| **IUGR** | 2/11 (18.18%) | 7/21 (33.33%) | 10/16(62.5%) | 8/22(40.90%) |
| **Postnatal growth failure** | 5/11(45.45%) | 8/21 (38.09%) | 11/16 (68.75%) | 9/22 (34.70%) |
| **Gestational week** | 39.95±1.38(40) | 38.13±2.15(39) | 37.140±3.5(38.5) | 38.73±1.8(39) |
| **Weight at birth** | 3006±436(3100) | 2735±785(2635) | 2505±93(2404) | 2483.50±566(2510) |
| **size at birth** | 46.45±5.88(48) | 46.06±3.23(46) | 44.30±3.83(44.9) | 46.44±3.29(47) |
| **OFC at birth** | 33.50±1.26(34) | 32.9±2.52(33) | 31.19±2.22(32.25) | 31.56±2.55(32) |
| **Postnatal** |  |  |  |  |
| **hypotonia** | 4/11 (36.36%) | 15/21 (71.42%) | 14/16(81.25%) | 16/22(72.27%) |
| **hypertonia** | 0/11(0%) | 4/21 (19.04%) | 1/16(6.25%) | 2/22 (9.09%) |
| **develpmental delay** | 11/11 (100.00%) | 19/21 (90.47%) | 15/16 (93.75%) | 19/22 (86.36%) |
| **light ID** | 4/11 (36.36%) | 3/21 (14.27%) | 0/16 (0%) | 1/22 (4.54%) |
| **moderate ID** | 3/11 (26.30%) | 6/21 (58.54%) | 3/16(18.75%) | 2/22 (9.09%) |
| **severe ID** | 0/11(0%) | 6/21 (58.54%) | 13/16(81.25%) | 12/22(54.54%) |
| **FUNCTIONAL** |  |  |  |  |
| **GFAP** | 366±106.77 (386) | 369.28±86.54(355) | 447.5±80.17(463) | 375.04±110.8(371) |
| ***Develop items*** | 223.36±57.9(220) | 237.76±48.77(234) | 280.56±54.44(279) | 235.59±84.67(244) |
| ***Behavioral alt .*** | 14.09±17.6(8) | 15.28±20.18(7) | 12.37±10.40(8.5) | 12.13±14.59(7.5) |
| ***Dismorphic feat.*** | 20.09±10.12(23) | 18.85±10.67(23) | 25.06±11.77(28.5) | 20.86±13.14(25) |
| ***Communication*** | 50.81±26.91(42) | 49.33±26.61(45) | 58.31±20.01(60) | 57.27±29.04(47.5) |
| ***Comorbidity*** | 57.63±38.50(44) | 48.04±37.02(48) | 71.18±48.23(69) | 49.18±39.36(42.5) |
| **COMORBIDITY FEATURES** |  |  |  |  |
| **MRI anomalies** | 6/11 (54.54%) | 7/16 (43.75%) | 13/16 (81.25%) | 17/22(72.27%) |
| **seizures** | 2/11 (18.18%) | 0/20 (0%) | 1/16(6.25%) | 1/22 (4.54%) |
| **high-pitched** | 4/11(36.36%) | 10/20(50%) | 10/16(62.5%) | 15/22(68.18%) |
| **cry w/o sound** | 0/11(0%) | 0/20 (0%) | 1/16(6.25%) | 1/22 (4.54%) |
| **breath difficulties** | 4/11 (36.36%) | 10/20(50%) | 8/16 (50%) | 4/22 (18.18%) |
| **cardiac anomalies** | 2/11 (18.18%) | 7/20 (35%) | 7/16 (43.75%) | 7/22 (31.81%) |
| **difficult to feed** | 6/11(54.54%) | 8/20 (40%) | 7/16 (43.75%) | 7/22 (31.81%) |
| **Laringyx and epiglotis alt.** | 3/11 (27.27%) | 16/21 (76.19%) | 6/16 (37.50%) | 7/22 (31.81%) |
| **gastrointest. alt.** | 6/11(54.54%) | 13/21 (61.90) | 10/16(62.5%) | 10/22 (45.45%) |
| **Renal anomalies** | 0/11(0%) | 4/21 (19.04%) | 2/16(12.50%) | 3/22 (13.63%) |
| **joint** | 7/11(63.63%) | 9/21 (42.85%) | 10/16(62.5%) | 10/22(45.45%) |
| **auditive problems** | 4/11(36.36%) | 11/21 (52.38%) | 9/16 (56.25%) | 6/22 (27.27%) |
| **ophtalmological prob.** | 3/11 (27.27%) | 10/21 (47.61%) | 7/16 (43.75%) | 12/22 (54.54%) |
| **genitalia anomalies** | 2/11 (18.18%) | 4/21 (19.04%) | 2/16 (12.50%) | 8/22(36.36%) |
| **sleeping problems** | 4/11 (36.36%) | 12/21 (57.14%) | 12/16 (75.00%) | 10/22(45.45%) |
| **SOCIAL** |  |  |  |  |
| **a familiy member quit job** | 2/11 (18.18%) | 9/21 (42.85%) | 9/16 (56.25%) | 12/22(54.54%) |
| **COGNITIVE** |  |  |  |  |
| **use diapers** | 2/11 (18.18%) | 10/21 (47.61%) | 9/16 (56.25%) | 10/22(45.45%) |
| **int with environment** | 9/11 (81.81%) | 14/21 (66.66%) | 12/16(75%) | 15/22 (68.18%) |
| **read/write** | 6/10(60%) | 4/20 (20.00%) | 1/16(6.25%) | 1/22 (4.54%) |
| **alternative comm syst** | 1/10(10%) | 8/20 (40.00%) | 11/16(68.75%) | 10/22 (45.45%) |
| **no words** | 1/10(10%) | 7/20 (35.00%) | 8/16 (50.00%) | 8/22 (36.36%) |
| **less than 10 words** | 5/10 (50%) | 5/20 (25.00%) | 5/16 (31.25%) | 10/22 (45.45%) |
| **sentences** | 6/10 (60%) | 5/20 (25.00%) | 2/16(12.50%) | 3/22 (13.63%) |
| **cephalic sosten** | 7/11(63.63%) | 14/21 (66.66%) | 13/16(81.25%) | 17/22(72.27%) |
| **MOTOR** |  |  |  |  |
| **stay seated** | 7/11(63.63%) | 13/21 (61.90%) | 11/16 (68.75%) | 17/22(72.27%) |
| **stay seated unaided** | 7/11(63.63%) | 11/21 (52.38%) | 11/16(68.75%) | 17/22(72.27%) |
| **walk unaided** | 9/11 (81.81%) | 12/21 (57.14%) | 7/16 (43.75%) | 13/22(59.09%) |
| **walK with help** | 8/11(72.72%) | 14/21 (66.66%) | 10/16(62.5%) | 15/22(68.18%) |
| **DISMORPHIC FEAT.** |  |  |  |  |
| **Microcephaly** | 10/11 (90.90%) | 17/21 (80.95%) | 15/16(93.75%) | 17/22(72.27%) |
| **facial assimetry** | 1/11 (9.09%) | 3/21 (14.28%) | 3/16(18.75%) | 2/22 (9.09%) |
| **round face** | 4/11 (36.36%) | 8/21 (38.095%) | 8/16(50.00%) | 12/22 (54.54%) |
| **enlarged face** | 4/11 (36.36%) | 8/21 (38.095%) | 7/16(43.75%) | 4/22 (18.18%) |
| **ear malformations** | 5/11 (45.45%) | 8/21 (38.095%) | 12/16(75.00%) | 13/22 (59.09%) |
| **epicanthus** | 1/11(9.09%) | 10/21 (47.62%) | 9/16 (56.25%) | 13/22(59.09%) |
| **hypertelorism** | 1/11(9.09% | 12/21(57.14%) | 12/16 (75.00%) | 16/22(71.00%) |
| **narrow nasal bridge** | 5/11 (45.45%) | 12/21 (57.14%) | 12/16 (75.00%) | 15/22(68.18%) |
| **short filtrum** | 3/11 (27.27%) | 2/21 (9.52%) | 3/16(18.75%) | 2/22(9.09%) |
| **cleft lip/palate, ojival** | 1/11 (9.09%) | 3/21 (14.28%) | 1/16(6.25%) | 2/22 (9.09%) |
| **micrognathia** | 1/11 (9.09%) | 9/21 (42.85%) | 8/16 (50.00%) | 12/22(54.54%) |
| **big mouth** | 0/11(0%) | 8/21 (38.09%) | 5/16(31.25%) | 5/22 (22.72%) |
| **Thick lower lip** | 1/11(9.09) | 4/21(19.04) | 5/16(31.25%) | 7/22(31.81) |
| **neck anomalies** | 3/11 (36.36%) | 2/21 (9.52%) | 4/16 (25.00%) | 4/22 (18.18%) |
| **teeth anomalies** | 3/11 (36.36%) | 10/21 (47.62%) | 10/16(62.5%) | 11/22(50.00%) |
| **downslanted palpebral fisures** | 2/11 (18.18%) | 1/21 (4.76%) | 5/16 (31.25%) | 6/22 (27.27%) |
| **BEHAVIOUR ALT.** | 10/11 (90.90%) | 14/21 (66.66%) | 11/16(68.75%) | 15/22(68.18%) |
| **autism** | 4/11 (36.36%) | 3/21 (14.28%) | 1/16 (6.25%) | 1/21 (4.54%) |
| **hyperactivity** | 4/11 (36.36%) | 5/21 (23.80%) | 4/16 (25.00%) | 4/22 (18.18%) |
| **agrressive** | 2/11 (18.18%) | 6/21 (28.57%) | 7/16(43.75%) | 12/22(54.54%) |
| **stereotypes** | 1/11 (9.09%) | 8/21 (38.09%) | 11/16(68.75%) | 11/22(50.00%) |
| **frustration** | 6/11(52.62%) | 6/21 (28.57%) | 7/16(43.75%) | 7/22 (31.81%) |
| **uncontrolled laughs** | 0/11(0%) | 6/21 (28.57%) | 7/16 (43.75%) | 7/22 (31.81%) |

See Figure 6C to be significant statistical differences between clusters pairs.
