# Supplementary material for: Deep Phenotyping and Genetic Characterization of a Cohort of 70 Individuals With 5p Minus Syndrome
Source: Front Genet. 2021 Jul 30;12:645595. doi: 10.3389/fgene.2021.645595 (PMC8362798; doi:10.3389/fgene.2021.645595)
Supplement: Supplementary file 7 [file Table_7.DOCX]

**Table 6. Supplemental data.** *Ward´s Cluster analysis (male vs female)*

| **Items** | **male** | **female** |
| --- | --- | --- |
| **size of deletion (Mb)** | 15.78±8.75 (15.02) range 0.62 -30.20 | 22.38±8.83 (25.00)*  range 4.35 -35.01 |
| **PRENATAL/NEONATAL** |  |  |
| **IUGR** | 3/23 (13.00%) | 24/47 (51.80%)* |
| **Postnatal growth failure** | 7/23 (30.40%) | 26/47 (51.10%)* |
| **Gestational week** | 38.51±2.19(39) | 38.18±2.77(39) |
| **Weight at birth** | 2925.91±689.24(2795) | 2447.28±621.32(2465)* |
| **Height at birth** | 47.11±3.78(47.25) | 45.30±3.86(46)^$^ |
| **OFC at birth** | 33.17±2.27(33.50) | 31.74±2.37(32)* |
| **POSTNATAL** |  |  |
| **hypotonia** | 14/23 (60.90%) | 35/47 (74.50%) |
| **hypertonia** | 1/23 (4.30%) | 6/47 (11.80%) |
| **developmental delay** | 21/23 (91.30%) | 43/47 (91.50%) |
| **light ID** | 5/23 (21.70%) | 3/47 (6.40%)^$^ |
| **moderate ID** | 7/23 (30.40%) | 7/47 (14.90%) |
| **severe ID** | 5/23 (21.70%) | 26/47 (55.30%)* |
| **FUNCTIONAL** |  |  |
| **GFAP** | 362.89±98.59(361.5) | 402.93±108.60(418)* |
| ***Developmental items*** | 231.22±48.51(236) | 251.15±73.22(269) |
| ***Behavioral alt .*** | 10.43±15.19(5) | 14.92±16.20(8) |
| ***Dysmorphic feat.*** | 20.09±10.31(24) | 21.60±12.35(26) |
| ***Communication*** | 53.35±26.80(45) | 54.49±25.72(50) |
| ***Comorbidity*** | 43.78±32.70(41) | 60.79±43.68(51) |
| **Comorbidity features** |  |  |
| **MRI anomalies** | 4/23 (17.40%) | 16/47 (34.00%) |
| **seizures** | 2/23 (8.70%) | 2/47 (4.30%) |
| **high-pitched cry** | 13/23 (56.50%) | 26/47(55.30%) |
| **cry w/o sound** | 0/23(0.00%) | 2/47 (4.30%) |
| **breathing difficulties** | 11/23 (47.80%) | 16/47 (34.00%) |
| **cardiac anomalies** | 5/23 (21.70%) | 19/47 (40.40% |
| **difficult to feed** | 8/23 (34.80%) | 20/47 (42.60% |
| **Laringyx and epiglottis alt.** | 5/23 (21.70%) | 18/47 (38.30% |
| **gastrointest. alt.** | 13/23 (56.50%) | 26/47(55.30%) |
| **Renal anomalies** | 2/23 (8.70%) | 7/47 (14.90%) |
| **hyperlaxity** | 10/23 (30.40%) | 21/47 (44.70%) |
| **auditory problems** | 13/23 (56.50%) | 16/47 (36.20% |
| **ophtalmological prob.** | 7/23 (25.53%) | 25/47 (53.20%) |
| **genitalia anomalies** | 6/23 (26.10%) | 10/47 (21.30%) |
| **scoliosis** | 4/23 (17.40%) | 21/47 (44.70%)* |
| **spinal anomalies** | 2/23 (8.70%) | 16/47 (34.00%)* |
| **sleeping problems** | 8/23 (34.80%) | 30/47 (63.80%)* |
| **SOCIAL** |  |  |
| **a family member quit job** | 10/23 (43.50%) | 22/47 (46.80%) |
| **COGNITIVE** |  |  |
| **use diapers** | 8/23 (34.80%) | 25/47 (48.90%) |
| **int with environment** | 18/23 (78.30%) | 32/47 (68.10%) |
| **Can read/write** | 7/23 (30.40%) | 5/45 (10.60%)* |
| **alternative comm syst** | 7/23 (30.40%) | 22/45 (46.80%) |
| **no words** | 9/23 (39.10%) | 5/45 (31.90%) |
| **less than 10 words** | 6/23 (26.10%) | 19/45 (40.40%)) |
| **sentences** | 9/23 (39.10%) | 7/45 (14.90%)* |
| **MOTOR** |  |  |
| **cephalic sosten** | 16/23 (69.60%) | 35/47 (74.50%) |
| **Able to stay seated** | 16/23 (69.60%) | 32/47 (68.10%) |
| **Able to stay seated unaided** | 15/23 (65.20%) | 31/47 (66.00%) |
| **walk unaided** | 15/23 (65.20%) | 26/47 (55.30%) |
| **walK with help** | 17/23 (73.90%) | 30/47 (63.80%) |
| **DYSMORPHIC FEAT.** |  |  |
| **microcephaly** | 21/23 (91.30%) | 38/47 (80.90%) |
| **facial assimetry** | 1/23 (4.30%) | 8/47 (17.00%) |
| **round face** | 6/23 (26.10%) | 26/47 (55.30%)* |
| **enlarged face** | 12/23 (52.20%) | 11/47 (23.40%)* |
| **ear malformations** | 13/23 (56.50%) | 25/47 (53.20%) |
| **epicanthus** | 10/23 (43.50%) | 23/47 (48.90%) |
| **hypertelorism** | 11/23 (47.80%) | 30/47 (63.80%) |
| **narrow nasal bridge** | 13/23 (56.50%) | 31/47 (66.00%) |
| **short philtrum** | 4/23 (17.40%) | 6/47 (12.80%) |
| **cleft lip/palate, ojival** | 2/23 (8.70%) | 5/47 (10.60%) |
| **micrognathia** | 8/23 (34.80%) | 22/47 (46.80%) |
| **big mouth** | 9/23 (39.10%) | 9/47 (19.10%) |
| **neck anomalies** | 4/23 (17.40%) | 12/47 (25.50%)* |
| **teeth anomalies** | 9/23 (39.10%) | 25/47 (53.20%) |
| **downslanted palpebral fisures** | 4/23 (17.40%) | 10/47 (21.30%) |
| **alterations of the fingers or toes** | 6/23 (26.10%) | 25/47 (53.20%)* |
| **BEHAVIOUR ALT.** | 16/23 (69.60%) | 16/23 (63.37%) |
| **ASD** | 4/23 (17.40%) | 5/47 (10.60%) |
| **hyperactivity** | 10/47 (21.27%) | 11/47 (23.40%) |
| **aggressive** | 4/23 (17.40%) | 23/47 (48.90%)* |
| **stereotypes** | 9/23 (39.10% | 22/47 (46.80%) |
| **Frustration intolerance** | 8/23 (34.80%)) | 18/47 (38.30%) |
| **uncontrolled laughs** | 5/23 (21.70%) | 15/47 (31.90%) |

* means significant differences among subpopulations (Chi square test)
