# Supplementary material for: Deep Phenotyping and Genetic Characterization of a Cohort of 70 Individuals With 5p Minus Syndrome
Source: Front Genet. 2021 Jul 30;12:645595. doi: 10.3389/fgene.2021.645595 (PMC8362798; doi:10.3389/fgene.2021.645595)
Supplement: Supplementary file 8 [file Table_8.docx]

**Table 7. Supplemental data.** Student-t test comparisons between males and females in all continuous variables in a) whole cohort, b) 5p deletions group c) 5p deletion + additional rearrangements.

1. Whole cohort

| **Test for independent samples** | | | | | | | | | | |
| --- | --- | --- | --- | --- | --- | --- | --- | --- | --- | --- |
|  | | Levene ´s test | | **Student- t test** | | | | | | |
|  |  | F | Sig. | t | gl | Sig. (bilateral) | Differences of means | Error standard differences | 95% CI | |
|  |  |  |  |  |  |  |  |  | Inferior | Superior |
| age at evaluation (years) | Variances are equals | ,933 | ,337 | -,377 | 68 | ,707 | -,8636 | 2,2896 | -5,4323 | 3,7052 |
|  | Variances are non-equals |  |  | -,419 | 57,408 | ,677 | -,8636 | 2,0630 | -4,9940 | 3,2669 |
| gestational age at birth (months) | Variances are equals | 1,542 | ,219 | ,474 | 66 | ,637 | ,3198 | ,6747 | -1,0273 | 1,6668 |
|  | Variances are non-equals |  |  | ,514 | 51,262 | ,609 | ,3198 | ,6217 | -,9282 | 1,5677 |
| weight at birth (gr) | Variances are equals | ,229 | ,634 | 2,868 | 66 | ,006* | 478,6265 | 166,8617 | 145,4764 | 811,7766 |
|  | Variances are non-equals |  |  | 2,764 | 37,829 | ,009 | 478,6265 | 173,1637 | 128,0228 | 829,2301 |
| height at birth (cm) | Variances are equals | ,001 | ,973 | 1,817 | 66 | ,074 | 1,80842 | ,99511 | -,17839 | 3,79523 |
|  | Variances are non-equals |  |  | 1,830 | 42,194 | ,074 | 1,80842 | ,98832 | -,18581 | 3,80265 |
| OFC at birth (cm) | Variances are equals | ,086 | ,771 | 2,365 | 66 | ,021* | 1,4334 | ,6060 | ,2235 | 2,6433 |
|  | Variances are non-equals |  |  | 2,401 | 43,103 | ,021 | 1,4334 | ,5969 | ,2298 | 2,6370 |
| size of deletion (Mb) | Variances are equals | ,042 | ,838 | -2,944 | 68 | ,004 | -6,598332 | 2,241583 | -11,071342 | -2,125322 |
|  | Variances are non-equals |  |  | -2,954 | 44,199 | ,005* | -6,598332 | 2,233496 | -11,099075 | -2,097589 |
| developmental delay corrected by age | Variances are equals | 4,013 | ,049 | -1,182 | 68 | ,241 | -19,9315 | 16,8575 | -53,5702 | 13,7071 |
|  | Variances are non-equals |  |  | -1,355 | 61,711 | ,180 | -19,9315 | 14,7110 | -49,3411 | 9,4781 |
| behavioral alterations | Variances are equals | ,104 | ,748 | -1,108 | 68 | ,272 | -4,4801 | 4,0433 | -12,5483 | 3,5881 |
|  | Variances are non-equals |  |  | -1,133 | 46,428 | ,263 | -4,4801 | 3,9538 | -12,4368 | 3,4766 |
| dysmorphic features | Variances are equals | ,923 | ,340 | -,506 | 68 | ,615 | -1,5088 | 2,9838 | -7,4628 | 4,4453 |
|  | Variances are non-equals |  |  | -,538 | 51,571 | ,593 | -1,5088 | 2,8041 | -7,1368 | 4,1192 |
| communication | Variances are equals | ,269 | ,605 | -,172 | 68 | ,864 | -1,1415 | 6,6363 | -14,3840 | 12,1009 |
|  | Variances are non-equals |  |  | -,170 | 42,200 | ,866 | -1,1415 | 6,7319 | -14,7252 | 12,4422 |
| Co-morbidity | Variances are equals | ,871 | ,354 | -1,652 | 68 | ,103 | -17,0046 | 10,2947 | -37,5474 | 3,5381 |
|  | Variances are non-equals |  |  | -1,822 | 56,570 | ,074 | -17,0046 | 9,3318 | -35,6943 | 1,6851 |
| global functional assessment of the patient (GFAP) | Variances are equals | 5,687 | ,020 | -1,756 | 68 | ,084 | -44,0666 | 25,0899 | -94,1327 | 5,9995 |
|  | Variances are non-equals |  |  | -2,001 | 60,942 | ,050* | -44,0666 | 22,0260 | -88,1112 | -,0220 |

*Means, p-value≤0.05

| **Group´s Statistics** | | | | | |
| --- | --- | --- | --- | --- | --- |
|  | sex | N | Mean | Standard deviation | Mean  Standard error |
| age at evaluation (years) | ,0 | 23 | 8,413 | 7,1651 | 1,4940 |
|  | 1,0 | 47 | 9,277 | 9,7529 | 1,4226 |
| gestational age at birth (months) | ,0 | 22 | 38,505 | 2,1975 | ,4685 |
|  | 1,0 | 46 | 38,185 | 2,7716 | ,4087 |
| Weight at birth (gr) | ,0 | 22 | 2925,909 | 689,2415 | 146,9468 |
|  | 1,0 | 46 | 2447,283 | 621,3261 | 91,6095 |
| Height at birth (cm) | ,0 | 22 | 47,1136 | 3,78887 | ,80779 |
|  | 1,0 | 46 | 45,3052 | 3,86204 | ,56943 |
| OFC at birth (cm) | ,0 | 22 | 33,168 | 2,2704 | ,4840 |
|  | 1,0 | 46 | 31,735 | 2,3686 | ,3492 |
| size of deletion (Mb) | ,0 | 23 | 15,78539 | 8,746937 | 1,823863 |
|  | 1,0 | 47 | 22,38372 | 8,838293 | 1,289198 |
| number of surgeries | ,0 | 23 | ,522 | 1,0388 | ,2166 |
|  | 1,0 | 47 | ,809 | 1,5128 | ,2207 |
| developmental delay corrected by age | ,0 | 23 | 231,217 | 48,5152 | 10,1161 |
|  | 1,0 | 47 | 251,149 | 73,2233 | 10,6807 |
| behavioral alterations | ,0 | 23 | 10,435 | 15,1982 | 3,1690 |
|  | 1,0 | 47 | 14,915 | 16,2090 | 2,3643 |
| dysmorphic features | ,0 | 23 | 20,087 | 10,3085 | 2,1495 |
|  | 1,0 | 47 | 21,596 | 12,3458 | 1,8008 |
| communication | ,0 | 23 | 53,348 | 26,8050 | 5,5892 |
|  | 1,0 | 47 | 54,489 | 25,7242 | 3,7523 |
| Co-morbidity | ,0 | 23 | 43,783 | 32,6983 | 6,8181 |
|  | 1,0 | 47 | 60,787 | 43,6811 | 6,3715 |
| global functional assessment of the patient (GFAP) | ,0 | 23 | 358,870 | 73,3933 | 15,3036 |
|  | 1,0 | 47 | 402,936 | 108,6023 | 15,8413 |

“1” means *“ever”* having a given condition compared to 0: *“never”* having the condition, taken from either of our two questionnaires’, and curated from medical records.

1. Simple 5p deletions

| **Test for independent samples** | | | | | | | | | | |
| --- | --- | --- | --- | --- | --- | --- | --- | --- | --- | --- |
|  | | Levene ´s test | | **Student- t test** | | | | | | |
|  |  | F | Sig. | t | gl | Sig. (bilateral) | Differences of means | Error standard differences | 95% CI | |
|  |  |  |  |  |  |  |  |  | Inferior | Superior |
| age at evaluation (years) | Variances are equals | 3,088 | ,086 | -1,096 | 41 | ,279 | -3,3171 | 3,0258 | -9,4279 | 2,7936 |
|  | Variances are non-equals |  |  | -1,341 | 35,930 | ,188 | -3,3171 | 2,4731 | -8,3332 | 1,6990 |
| gestational age at birth (months) | Variances are equals | ,042 | ,838 | -,294 | 39 | ,771 | -,2254 | ,7674 | -1,7777 | 1,3269 |
|  | Variances are non-equals |  |  | -,293 | 29,034 | ,772 | -,2254 | ,7701 | -1,8004 | 1,3496 |
| Weight at birth (gr) | Variances are equals | ,031 | ,862 | 1,495 | 39 | ,143 | 314,5513 | 210,3402 | -110,9019 | 740,0045 |
|  | Variances are non-equals |  |  | 1,511 | 30,303 | ,141 | 314,5513 | 208,1070 | -110,2816 | 739,3841 |
| height at birth (cm) | Variances are equals | ,045 | ,834 | ,959 | 39 | ,344 | 1,16641 | 1,21680 | -1,29479 | 3,62761 |
|  | Variances are non-equals |  |  | ,949 | 28,465 | ,351 | 1,16641 | 1,22900 | -1,34924 | 3,68206 |
| OFC at birth (cm) | Variances are equals | ,130 | ,720 | ,571 | 39 | ,571 | ,4518 | ,7907 | -1,1475 | 2,0511 |
|  | Variances are non-equals |  |  | ,597 | 33,331 | ,555 | ,4518 | ,7567 | -1,0872 | 1,9908 |
| number of surgeries | Variances are equals | 3,607 | ,065 | -,910 | 41 | ,368 | -,4144 | ,4552 | -1,3336 | ,5049 |
|  | Variances are non-equals |  |  | -1,074 | 39,686 | ,289 | -,4144 | ,3858 | -1,1942 | ,3655 |
| size of deletion (Mb) | Variances are equals | ,000 | ,990 | -1,697 | 41 | ,097 | -4,755144 | 2,801343 | -10,412570 | ,902283 |
|  | Variances are non-equals |  |  | -1,724 | 33,155 | ,094 | -4,755144 | 2,758733 | -10,366832 | ,856545 |
| developmental delay corrected by age | Variances are equals | 4,129 | ,049 | -1,312 | 41 | ,197 | -26,3356 | 20,0800 | -66,8881 | 14,2168 |
|  | Variances are non-equals |  |  | -1,442 | 40,008 | ,157 | -26,3356 | 18,2634 | -63,2471 | 10,5758 |
| behavioral alteration | Variances are equals | 2,686 | ,109 | -1,914 | 41 | ,063 | -8,5139 | 4,4477 | -17,4962 | ,4685 |
|  | Variances are non-equals |  |  | -2,049 | 38,143 | ,047 | -8,5139 | 4,1546 | -16,9235 | -,1043 |
| dysmorphic features | Variances are equals | ,262 | ,612 | -,978 | 41 | ,334 | -4,0046 | 4,0952 | -12,2751 | 4,2659 |
|  | Variances are non-equals |  |  | -1,025 | 36,249 | ,312 | -4,0046 | 3,9076 | -11,9277 | 3,9185 |
| Communication skills | Variances are equals | ,368 | ,547 | ,360 | 41 | ,721 | 3,0787 | 8,5491 | -14,1866 | 20,3440 |
|  | Variances are non-equals |  |  | ,356 | 30,432 | ,725 | 3,0787 | 8,6555 | -14,5878 | 20,7452 |
| Co-morbidity items | Variances are equals | 3,589 | ,065 | -1,769 | 41 | ,084 | -24,2824 | 13,7302 | -52,0111 | 3,4463 |
|  | Variances are non-equals |  |  | -2,085 | 39,761 | ,044 | -24,2824 | 11,6488 | -47,8300 | -,7349 |
| global functional assessment of the patient (GFAP) | Variances are equals | 10,461 | ,002 | -1,816 | 41 | ,077 | -60,0579 | 33,0703 | -126,8447 | 6,7289 |
|  | Variances are non-equals |  |  | -2,128 | 40,141 | ,040* | -60,0579 | 28,2234 | -117,0933 | -3,0224 |

*Means, p-value≤0.05

| **Group´s Statistics** | | | | | |
| --- | --- | --- | --- | --- | --- |
|  | sex | N | Media | Desviación estándar | Media de error estándar |
| age at evaluation (years) | ,0 | 16 | 8,313 | 4,2539 | 1,0635 |
|  | 1,0 | 27 | 11,630 | 11,6020 | 2,2328 |
| gestational age at birth (months) | ,0 | 15 | 38,140 | 2,3862 | ,6161 |
|  | 1,0 | 26 | 38,365 | 2,3561 | ,4621 |
| Weight at birth (gr) | ,0 | 15 | 2744,667 | 632,5670 | 163,3281 |
|  | 1,0 | 26 | 2430,115 | 657,6047 | 128,9669 |
| Hight at birth (cm) | ,0 | 15 | 46,7333 | 3,83995 | ,99147 |
|  | 1,0 | 26 | 45,5669 | 3,70314 | ,72625 |
| OFC at birth (cm) | ,0 | 15 | 32,413 | 2,1869 | ,5647 |
|  | 1,0 | 26 | 31,962 | 2,5687 | ,5038 |
| number of surgeries | ,0 | 16 | ,438 | ,8139 | ,2035 |
|  | 1,0 | 27 | ,852 | 1,7030 | ,3277 |
| size of deletion (Mb) | ,0 | 16 | 18,20838 | 8,548711 | 2,137178 |
|  | 1,0 | 27 | 22,96352 | 9,064389 | 1,744443 |
| developmental delay corrected by age | ,0 | 16 | 221,813 | 48,5527 | 12,1382 |
|  | 1,0 | 27 | 248,148 | 70,9071 | 13,6461 |
| behavioral alterations | ,0 | 16 | 7,375 | 11,7239 | 2,9310 |
|  | 1,0 | 27 | 15,889 | 15,3004 | 2,9446 |
| dysmorphic features | ,0 | 16 | 18,625 | 11,4884 | 2,8721 |
|  | 1,0 | 27 | 22,630 | 13,7677 | 2,6496 |
| communication | ,0 | 16 | 52,375 | 27,9067 | 6,9767 |
|  | 1,0 | 27 | 49,296 | 26,6196 | 5,1229 |
| Co-morbidity | ,0 | 16 | 36,125 | 24,6924 | 6,1731 |
|  | 1,0 | 27 | 60,407 | 51,3309 | 9,8786 |
| global functional assessment of the patient (GFAP) | ,0 | 16 | 336,313 | 61,3577 | 15,3394 |
|  | 1,0 | 27 | 396,370 | 123,1023 | 23,6910 |

“1” means *“ever”* having a given condition compared to 0: *“never”* having the condition, taken from either of our two questionnaires’, and curated from medical records.

1. 5p deletions + additional rearrangements

| **Test for independent samples** | | | | | | | | | | |
| --- | --- | --- | --- | --- | --- | --- | --- | --- | --- | --- |
|  | | Levene ´s test | | **Student- t test** | | | | | | |
|  |  | F | Sig. | t | gl | Sig. (bilateral) | Differences of means | Error standard differences | 95% CI | |
|  |  |  |  |  |  |  |  |  | Inferior | Superior |
| age at evaluation (years) | Variances are equals | 2,518 | ,125 | ,779 | 25 | ,443 | 2,5429 | 3,2649 | -4,1814 | 9,2671 |
|  | Variances are non-equals |  |  | ,545 | 6,829 | ,603 | 2,5429 | 4,6686 | -8,5531 | 13,6388 |
| gestational age at birth (months) | Variances are equals | 3,501 | ,073 | 1,024 | 25 | ,315 | 1,3357 | 1,3039 | -1,3496 | 4,0211 |
|  | Variances are non-equals |  |  | 1,403 | 21,748 | ,175 | 1,3357 | ,9522 | -,6404 | 3,3118 |
| weight at birth (gr) | Variances are equals | ,247 | ,624 | 3,142 | 25 | ,004* | 844,6857 | 268,8393 | 291,0009 | 1398,3705 |
|  | Variances are non-equals |  |  | 2,906 | 9,273 | ,017 | 844,6857 | 290,6800 | 190,0576 | 1499,3138 |
| height at birth (cm) | Variances are equals | ,012 | ,913 | 1,661 | 25 | ,109 | 2,96357 | 1,78381 | -,71026 | 6,63740 |
|  | Variances are non-equals |  |  | 1,724 | 11,278 | ,112 | 2,96357 | 1,71862 | -,80775 | 6,73490 |
| OFC at birth (cm) | Variances are equals | ,330 | ,571 | 3,831 | 25 | ,001* | 3,3457 | ,8733 | 1,5472 | 5,1443 |
|  | Variances are non-equals |  |  | 4,448 | 14,372 | ,001 | 3,3457 | ,7521 | 1,7364 | 4,9550 |
| number of surgeries | Variances are equals | ,016 | ,901 | -,062 | 25 | ,951 | -,0357 | ,5771 | -1,2244 | 1,1529 |
|  | Variances are non-equals |  |  | -,057 | 9,125 | ,956 | -,0357 | ,6309 | -1,4599 | 1,3885 |
| size of deletion (Mb) | Variances are equals | ,165 | ,688 | -3,122 | 25 | ,004* | -11,353857 | 3,636340 | -18,843039 | -3,864675 |
|  | Variances are non-equals |  |  | -3,520 | 13,414 | ,004 | -11,353857 | 3,225779 | -18,300905 | -4,406810 |
| developmental delay corrected by age | Variances are equals | 1,053 | ,315 | -,079 | 25 | ,937 | -2,4857 | 31,3143 | -66,9786 | 62,0072 |
|  | Variances are non-equals |  |  | -,103 | 18,997 | ,919 | -2,4857 | 24,1620 | -53,0579 | 48,0865 |
| behavioral alterations | Variances are equals | 1,114 | ,301 | ,474 | 25 | ,640 | 3,8286 | 8,0817 | -12,8159 | 20,4730 |
|  | Variances are non-equals |  |  | ,440 | 9,322 | ,670 | 3,8286 | 8,7070 | -15,7648 | 23,4219 |
| dysmorphic features | Variances are equals | 5,145 | ,032 | ,773 | 25 | ,447 | 3,2286 | 4,1763 | -5,3726 | 11,8298 |
|  | Variances are non-equals |  |  | ,967 | 17,315 | ,347 | 3,2286 | 3,3397 | -3,8078 | 10,2649 |
| Communication skills | Variances are equals | ,232 | ,634 | -,563 | 25 | ,579 | -5,9286 | 10,5327 | -27,6210 | 15,7638 |
|  | Variances are non-equals |  |  | -,532 | 9,586 | ,607 | -5,9286 | 11,1427 | -30,9023 | 19,0451 |
| Co-morbidit yitems | Variances are equals | 1,538 | ,226 | -,001 | 25 | ,999 | -,0143 | 15,3510 | -31,6303 | 31,6017 |
|  | Variances are non-equals |  |  | -,001 | 8,380 | ,999 | -,0143 | 17,8762 | -40,9136 | 40,8850 |
| global functional assessment of the patient (GFAP) | Variances are equals | ,061 | ,806 | -,037 | 25 | ,971 | -1,3714 | 37,3757 | -78,3481 | 75,6052 |
|  | Variances are non-equals |  |  | -,039 | 11,945 | ,969 | -1,3714 | 34,9825 | -77,6304 | 74,8876 |

*Means, p-value≤0.05

| **Statistics** | | | | | |
| --- | --- | --- | --- | --- | --- |
|  | sex | N | Mean | Sd | Mean Standard error |
| age at evaluation (years) | ,0 | 7 | 8,643 | 11,9546 | 4,5184 |
|  | 1,0 | 20 | 6,100 | 5,2534 | 1,1747 |
| gestational age at birth (months) | ,0 | 7 | 39,286 | 1,6036 | ,6061 |
|  | 1,0 | 20 | 37,950 | 3,2843 | ,7344 |
| Weight at birth (gr) | ,0 | 7 | 3314,286 | 686,2424 | 259,3752 |
|  | 1,0 | 20 | 2469,600 | 586,8449 | 131,2225 |
| height at birth (cm) | ,0 | 7 | 47,9286 | 3,83437 | 1,44926 |
|  | 1,0 | 20 | 44,9650 | 4,13117 | ,92376 |
| OFC at birth (cm) | ,0 | 7 | 34,786 | 1,5507 | ,5861 |
|  | 1,0 | 20 | 31,440 | 2,1080 | ,4714 |
| number of surgeries | ,0 | 7 | ,714 | 1,4960 | ,5654 |
|  | 1,0 | 20 | ,750 | 1,2513 | ,2798 |
| size of deletion (Mb) | ,0 | 7 | 10,24714 | 6,811147 | 2,574372 |
|  | 1,0 | 20 | 21,60100 | 8,692822 | 1,943774 |
| developmental delay corrected by age | ,0 | 7 | 252,714 | 44,2934 | 16,7413 |
|  | 1,0 | 20 | 255,200 | 77,9140 | 17,4221 |
| behavioral alterations | ,0 | 7 | 17,429 | 20,5252 | 7,7578 |
|  | 1,0 | 20 | 13,600 | 17,6796 | 3,9533 |
| dysmorphic features | ,0 | 7 | 23,429 | 6,3994 | 2,4187 |
|  | 1,0 | 20 | 20,200 | 10,2987 | 2,3029 |
| Communication skills | ,0 | 7 | 55,571 | 26,0631 | 9,8509 |
|  | 1,0 | 20 | 61,500 | 23,2888 | 5,2075 |
| Co-morbidity items | ,0 | 7 | 61,286 | 43,3848 | 16,3979 |
|  | 1,0 | 20 | 61,300 | 31,8336 | 7,1182 |
| global functional assessment of the patient (GFAP) | ,0 | 7 | 410,429 | 76,6852 | 28,9843 |
|  | 1,0 | 20 | 411,800 | 87,5999 | 19,5879 |

“1” means *“ever”* having a given condition compared to 0: *“never”* having the condition, taken from either of our two questionnaires’, and curated from medical records.
