# Supplementary material for: Deep Phenotyping and Genetic Characterization of a Cohort of 70 Individuals With 5p Minus Syndrome
Source: Front Genet. 2021 Jul 30;12:645595. doi: 10.3389/fgene.2021.645595 (PMC8362798; doi:10.3389/fgene.2021.645595)
Supplement: Supplementary file 9 [file Table_9.DOCX]

**Table 9. Supplemental Data*.*** *Genes located at the short arm of chromosome-5 hypothetically linked to 5p- syndrome…..*

| Name | localization | Description | %HI^#^ | pLI^&^ |
| --- | --- | --- | --- | --- |
| *PLEKHG4B* | 5:140373-190085 | pleckstrin homology and RhoGEF domain containing G4B | 90.04 | * |
| *LRRC14B* | 5:191626-195468 | leucine rich repeat containing 14B | 76.34 | * |
| *CCDC127* | 5:196986-218330 | coiled-coil domain containing 127 | 54.88 | 0.02* |
| *SDHA* | 5:218356-256815 | succinate dehydrogenase complex flavoprotein subunit A | 49.09 | * |
| *PDCD6* | 5:271736-353971 | programmed cell death 6 | 46.79 | 0.71* |
| *AHRR* | 5:304291-438406 | aryl-hydrocarbon receptor repressor | 86.99 | * |
| *C5orf55* | 5:441645-443258 | EXOC3 antisense RNA 1 | 96.33 | 0.05* |
| *EXOC3* | 5:443273-472052 | exocyst complex component 3 | 48.57 | 0.76 |
| *SLC9A3* | 5:473425-524447 | solute carrier family 9 member A3 | 62.15 | **0.99** |
| *MIR4456* | 5:535955-535997 | microRNA 4456 | * | * |
| *CEP72* | 5:612387-667283 | centrosomal protein 72 | 83.88 | * |
| *TPPP* | 5:660883-693510 | tubulin polymerization promoting protein | 52.83 | 0.45 |
| *ZDHHC11* | 5:710471-851101 | zinc finger DHHC-type containing 11 | 98.53 | * |
| *ZDHHC11B* | 5:710475-767067 | zinc finger DHHC-type containing 11B | 98.37 | 0.04* |
| *SPCS2P3* | 5:816461-817116 | signal peptidase complex subunit 2 pseudogene 3 | * | * |
| *BRD9* | 5:850406-892939 | bromodomain containing 9 | 69.00 | 0.25 |
| *TRIP13* | 5:892758-919472 | thyroid hormone receptor interactor 13 | **36.57** | **0.97** |
| *NKD2* | 5:1008944-1039058 | NKD inhibitor of WNT signaling pathway 2 | 84.18 | * |
| *SLC12A7* | 5:1050499-1112150 | solute carrier family 12 member 7 | 70.30 | * |
| *MIR4635* | 5:1063011-1063089 | microRNA 4635 | * | * |
| *SLC6A19* | 5:1201710-1225232 | solute carrier family 6 member 19 | **41.72** | * |
| *SLC6A18* | 5:1225470-1246304 | solute carrier family 6 member 18 | 69.66 | * |
| *TERT* | 5:1253262-1295184 | telomerase reverse transcriptase | **18.56** | **0.87** |
| *MIR4457* | 5:1309425-1309492 | microRNA 4457 | * | * |
| *CLPTM1L* | 5:1317859-1345214 | CLPTM1 like | 57.25 | 0.31 |
| *SLC6A3* | 5:1392909-1445545 | solute carrier family 6 member 3 | **18.92** | **1.00** |
| *LPCAT1* | 5:1456595-1524092 | lysophosphatidylcholine acyltransferase 1 | 75.72 | 0.81 |
| *SDHAP3* | 5:1568637-1594735 | succinate dehydrogenase complex flavoprotein subunit A pseudogene 3 | * | * |
| *MIR4277* | 5:1708900-1708983 | microRNA 4277 | * | * |
| *MRPL36* | 5:1798500-1801480 | mitochondrial ribosomal protein L36 | 90.97 | 0.25 |
| *NDUFS6* | 5:1801514-1816719 | NADH:ubiquinone oxidoreductase subunit S6 | 81.81 | * |
| *IRX4* | 5:1877541-1887350 | iroquois homeobox 4 | 62.07 | 0.25 |
| *IRX2* | 5:2745959-2752969 | iroquois homeobox 2 | **34.30** | **0.65** |
| *C5orf38* | 5:2752245-2755508 | chromosome 5 open reading frame 38 | 81.61 | 0.16 |
| *LINC01019* | 5:3417266-3536208 | long intergenic non-protein coding RNA 1019 | * | * |
| *IRX1* | 5:3596168-3601517 | iroquois homeobox 1 | **30.53** | * |
| *LINC01020* | 5:5034472-5070117 | long intergenic non-protein coding RNA 1020 | * | * |
| *RN7SKP73* | 5:5126159-5126450 | RN7SK pseudogene 73 | * | * |
| *ADAMTS16* | 5:5140443-5320417 | ADAM metallopeptidase with thrombospondin type 1 motif 16 | 75.39 | * |
| *ALG3P1* | 5:5375836-5376949 | ALG3 pseudogene 1 | * | * |
| *KIAA0947* | 5:5420777-5490347 | interactor of little elongation complex ELL subunit 1 | 92.65 | **1.00** |
| *HMGB3P3* | 5:6289970-6290737 | high mobility group box 3 pseudogene 3 | * | * |
| *MED10* | 5:6371994-6378707 | mediator complex subunit 10 | **24.48** | 0.04* |
| *UBE2QL1* | 5:6448736-6495022 | ubiquitin conjugating enzyme E2 Q family like 1 | 43.73 | * |
| *LINC01018* | 5:6582249-6588612 | long intergenic non-protein coding RNA 1018 | * | * |
| *NSUN2* | 5:6599352-6633404 | NOP2/Sun RNA methyltransferase 2 | 66.65 | **0.96** |
| *SRD5A1* | 5:6633456-6669675 | steroid 5 alpha-reductase 1 | 86.50 | 0.00 |
| *PAPD7* | 5:6714718-6757161 | terminal nucleotidyltransferase 4A | **44.90** | **1.00** |
| *RN7SKP79* | 5:6848240-6848575 | RN7SK pseudogene 79 | * | * |
| *RNA5SP176* | 5:7047340-7047449 | RNA, 5S ribosomal pseudogene 176 | * | * |
| *ADCY2* | 5:7396321-7830194 | adenylate cyclase 2 | **3.34** | **1.00** |
| *C5orf49* | 5:7830491-7851603 | chromosome 5 open reading frame 49 | 76.86 | 0.35 |
| *MTRR* | 5:7851299-7906138 | 5-methyltetrahydrofolate-homocysteine methyltransferase reductase | 73.75 | * |
| *FASTKD3* | 5:7859272-7869150 | FAST kinase domains 3 | 90.64 | * |
| *RNU1-76P* | 5:7980259-7980417 | RNA, U1 small nuclear 76, pseudogene | * | * |
| *MIR4458HG* | 5:8450856-8463208 | MIR4458 host gene | * | * |
| *MTND6P2* | 5:8618693-8619216 | MT-ND6 pseudogene 2 | * | * |
| *SEMA5A* | 5:9035138-9546187 | semaphorin 5A | **26.39** | * |
| *MIR4636* | 5:9053928-9054007 | microRNA 4636 | * | * |
| *SNHG18* | 5:9546312-9550721 | small nucleolar RNA host gene 18 | * | * |
| *SNORD123* | 5:9548948-9549017 | small nucleolar RNA, C/D box 123 | * | * |
| *TAS2R1* | 5:9629109-9712490 | taste 2 receptor member 1 | 99.90 | 0.24 |
| *RNA5SP177* | 5:9761019-9761125 | RNA, 5S ribosomal pseudogene 177 | * | * |
| *FAM173B* | 5:10226442-10250009 | ATP synthase c subunit lysine N-methyltransferase | 87.49 | * |
| *CCT5* | 5:10250033-10266524 | chaperonin containing TCP1 subunit 5 | **23.04** | **1.00** |
| *CMBL* | 5:10275987-10308138 | carboxymethylenebutenolidase homolog | 72.13 | 0.01* |
| *MARCH6* | 5:10353815-10440500 | membrane associated ring-CH-type finger 6 | **33.87** | **1.00** |
| *ROPN1L-AS1* | 5:10441402-10441904 | ROPN1L antisense RNA 1 | * | * |
| *ROPN1L* | 5:10441636-10472141 | rhophilin associated tail protein 1 like | 80.47 | 0.00 |
| *RPL30P7* | 5:10488932-10489254 | ribosomal protein L30 pseudogene 7 | * | * |
| *ANKRD33B* | 5:10564442-10650308 | ankyrin repeat domain 33B | 76.38 | * |
| *ANKRD33B-AS1* | 5:10627372-10628337 | ANKRD33B antisense RNA 1 | * | * |
| *DAP* | 5:10679342-10761384 | death associated protein | 71.27 | 0.00 |
| *CTNND2* | 5:10971952-11904155 | catenin delta 2 | **5.83** | **1.00** |
| *RNU6-429P* | 5:11027312-11027419 | RNA, U6 small nuclear 429, pseudogene | * | * |
| *RNU6-679P* | 5:12297511-12297616 | RNA, U6 small nuclear 679, pseudogene | * | * |
| *CT49* | 5:12574969-12804475 | long intergenic non-protein coding RNA 1194 | * | * |
| *RPS23P5* | 5:13250347-13250774 | ribosomal protein S23 pseudogene 5 | * | * |
| *DNAH5* | 5:13690440-13944652 | dynein axonemal heavy chain 5 | **32.91** | * |
| *TRIO* | 5:14143811-14532235 | trio Rho guanine nucleotide exchange factor | **25.69** | **1.00** |
| *FAM105A* | 5:14581884-14615116 | OTU deubiquitinase with linear linkage specificity like | 60.84 | * |
| *CCT6P2* | 5:14639535-14641127 | chaperonin containing TCP1 subunit 6 pseudogene 2 | * | * |
| *EEF1A1P13* | 5:14652047-14653438 | eukaryotic translation elongation factor 1 alpha 1 pseudogene 13 | * | * |
| *FAM105B* | 5:14664773-14699820 | OTU deubiquitinase with linear linkage specificity | **46.75** | **0.97** |
| *ANKH* | 5:14704910-14871887 | ANKH inorganic pyrophosphate transport regulator | **40.29** | 0.17 |
| *RBBP4P1* | 5:14797234-14798509 | RBBP4 pseudogene 1 | * | * |
| *MIR4637* | 5:14826038-14826121 | microRNA 4637 | * | * |
| *UQCRBP3* | 5:14874509-14874820 | ubiquinol-cytochrome c reductase binding protein pseudogene 3 | * | * |
| *HNRNPKP5* | 5:14877776-14878028 | heterogeneous nuclear ribonucleoprotein K pseudogene 5 | * | * |
| *SEPHS2P1* | 5:14960341-14961502 | selenophosphate synthetase 2 pseudogene 1 | * | * |
| *MARK2P5* | 5:15384329-15384962 | microtubule affinity regulating kinase 2 pseudogene 5 | * | * |
| *FBXL7* | 5:15500305-15939900 | F-box and leucine rich repeat protein 7 | **15.65** | 0.20 |
| *MIR887* | 5:15935291-15935369 | microRNA 887 | * | * |
| *RNA5SP178* | 5:16033338-16033465 | RNA, 5S ribosomal pseudogene 178 | * | * |
| *MARCH11* | 5:16067248-16180871 | membrane associated ring-CH-type finger 11 | **38.14** | * |
| *NACAP6* | 5:16192197-16192818 | NACA pseudogene 6 | * | * |
| *ZNF622* | 5:16451628-16465901 | zinc finger protein 622 | 54.00 | 0.09 |
| *FAM134B* | 5:16473147-16617167 | reticulophagy regulator 1 | 47.63 | 0.01 |
| *MYO10* | 5:16665395-16936372 | myosin X | 44.15 | * |
| *RNA5SP179* | 5:16854103-16854223 | RNA, 5S ribosomal pseudogene 179 | * | * |
| *RNU6-660P* | 5:16941952-16942060 | RNA, U6 small nuclear 660, pseudogene | * | * |
| *BASP1* | 5:17065707-17276943 | brain abundant membrane attached signal protein 1 | 55.71 | 0.54 |
| *RNA5SP180* | 5:17157080-17157180 | RNA, 5S ribosomal pseudogene 180 | * | * |
| *DCAF13P2* | 5:17202383-17203254 | DDB1 and CUL4 associated factor 13 pseudogene 2 | * | * |
| *RNU6-1003P* | 5:17240720-17240824 | RNA, U6 small nuclear 1003, pseudogene | * | * |
| *RN7SKP133* | 5:17345725-17346064 | RN7SK pseudogene 133 | * | * |
| *FTH1P10* | 5:17353804-17354733 | ferritin heavy chain 1 pseudogene 10 | * | * |
| *RPL36AP21* | 5:18049659-18049981 | ribosomal protein L36a pseudogene 21 | * | * |
| *RN7SL58P* | 5:18547421-18547712 | RNA, 7SL, cytoplasmic 58, pseudogene | * | * |
| *UBE2V1P12* | 5:18886731-18887204 | ubiquitin conjugating enzyme E2 V1 pseudogene 12 | * | * |
| *RPL32P14* | 5:19041118-19041495 | ribosomal protein L32 pseudogene 14 | * | * |
| *HSPD1P15* | 5:19233475-19234596 | heat shock protein family D (Hsp60) member 1 pseudogene 15 | * | * |
| *CDH18* | 5:19473060-20575982 | cadherin 18 | **14.09** | **0.46** |
| *GUSBP1* | 5:21341942-21589481 | GUSB pseudogene 1 | * | * |
| *CDH12* | 5:21750782-22853731 | cadherin 12 | **12.98** | * |
| *HSPD1P1* | 5:21882694-21884421 | heat shock protein family D (Hsp60) member 1 pseudogene 1 | * | * |
| *PMCHL1* | 5:22142461-22152465 | pro-melanin concentrating hormone like 1 (pseudogene) | * | * |
| *GCNT1P2* | 5:22580334-22580928 | glucosaminyl (N-acetyl) transferase 1, core 2 pseudogene 2 | * | * |
| *RN7SL572P* | 5:23201554-23201836 |  | * | * |
| *PRDM9* | 5:23507264-23528706 | PR/SET domain 9 | 84.03 | * |
| *C5orf17* | 5:23951457-24178372 | chromosome 5 open reading frame 17 (putative) | 92.73 | * |
| *CDH10* | 5:24487209-24645087 | cadherin 10 | **21.10** | 0.31 |
| *RNU6-374P* | 5:25701326-25701433 | RNA, U6 small nuclear 374, pseudogene | * | * |
| *MSNP1* | 5:25909612-25911343 | moesin pseudogene 1 | * | * |
| *RNU4-43P* | 5:26012998-26013134 | RNA, U4 small nuclear 43, pseudogene | * | * |
| *CDH9* | 5:26880709-27121257 | cadherin 9 | **34.45** | **0.99** |
| *RNU6-738P* | 5:27111676-27111781 | RNA, U6 small nuclear 738, pseudogene | * | * |
| *LINC01021* | 5:27472399-27496508 | p53 upregulated regulator of p53 levels | * | * |
| *RNU6-909P* | 5:28624764-28624870 | RNA, U6 small nuclear 909, pseudogene | * | * |
| *LSP1P3* | 5:28925707-28927696 | LSP1 pseudogene 3 | * | * |
| *SUCLG2P4* | 5:29001867-29003464 | succinate-CoA ligase GDP-forming beta subunit pseudogene 4 | * | * |
| *UBL5P1* | 5:29600783-29600975 | ubiquitin like 5 pseudogene 1 | * | * |
| *RN7SKP207* | 5:29948935-29949237 | RN7SK pseudogene 207 | * | * |
| *RPL19P11* | 5:31053672-31054260 | ribosomal protein L19 pseudogene 11 | * | * |
| *CDH6* | 5:31193857-31329253 | cadherin 6 | **5.78** | **0.98** |
| *DROSHA* | 5:31400604-31532303 | drosha ribonuclease III | **7.70** | **1.00** |
| *C5orf22* | 5:31532373-31555165 | chromosome 5 open eading frame 22 | 60.33 | 0.17 |
| *RNU6-363P* | 5:31595672-31595776 | RNA, U6 small nuclear 363, pseudogene | * | * |
| *PDZD2* | 5:31639517-32111037 | PDZ domain containing 2 | 71.80 | **1.00** |
| *RNU6-358P* | 5:31820671-31820745 | RNA, U6 small nuclear 358, pseudogene | * | * |
| *RNU6-760P* | 5:31832546-31832619 | RNA, U6 small nuclear 760, pseudogene | * | * |
| *RPL5P14* | 5:31847377-31848254 | ribosomal protein L5 pseudogene 14 | * | * |
| *TPT1P5* | 5:31908467-31909292 | tumor protein, translationally-controlled 1 pseudogene 5 | * | * |
| *MIR4279* | 5:31936208-31936265 | microRNA 4279 | * | * |
| *GOLPH3* | 5:32124810-32174456 | golgi phosphoprotein 3 | **7.00** | 0.01* |
| *MTMR12* | 5:32227100-32313115 | myotubularin related protein 12 | **41.79** | **1.00** |
| *RNU6-1079P* | 5:32234872-32234981 | RNA, U6 small nuclear 1079, pseudogene | * | * |
| *RNU6-378P* | 5:32309768-32309874 | RNA, U6 small nuclear 378, pseudogene | * | * |
| *ZFR* | 5:32354456-32444867 | zinc finger RNA binding protein | **9.24** | **1.00** |
| *MIR579* | 5:32394484-32394581 | microRNA 579 | * | * |
| *SUB1* | 5:32531739-32604185 | SUB1 regulator of transcription | **11.34** | **0.80** |
| *NPR3* | 5:32689176-32791819 | natriuretic peptide receptor 3 | **26.99** | * |
| *TRAS* | 5:33440802-33469644 | threonyl-tRNA synthetase | **15.32** | * |
| *ADAMTS12* | 5:33523640-33892297 | ADAM metallopeptidase with thrombospondin type 1 motif 12 | 53.40 | * |
| *RNU6-923P* | 5:33888161-33888267 | RNA, U6 small nuclear 923, pseudogene | * | * |
| *RXFP3* | 5:33936491-33939023 | relaxin family peptide receptor 3 | 47.85 | * |
| *SLC45A2* | 5:33944721-33984835 | solute carrier family 45 member 2 | 42.10 | * |
| *AMACR* | 5:33986283-34008220 | alpha-methylacyl-CoA racemase | 66.33 | 0.13 |
| *C1QTNF3* | 5:34019553-34043937 | C1q and TNF related 3 | **27.12** | * |
| *RAI14* | 5:34656342-34832732 | retinoic acid induced 14 | **22.65** | **0.49** |
| *TTC23L* | 5:34838938-34899561 | tetratricopeptide repeat domain 23 like | 66.47 | * |
| *RPL21P54* | 5:34883162-34883625 | ribosomal protein L21 pseudogene 54 | * | * |
| *RAD1* | 5:34905369-34919094 | RAD1 checkpoint DNA exonuclease | **22.50** | * |
| *BRIX1* | 5:34915481-34926101 | biogenesis of ribosomes BRX1 | **6.52** | **0.98** |
| *DNAJC21* | 5:34929698-34959069 | DnaJ heat shock protein family (Hsp40) member C21 | 49.27 | * |
| *AGXT2* | 5:34998206-35048198 | alanine--glyoxylate aminotransferase 2 | 50.18 | * |
| *PRLR* | 5:35048861-35230794 | prolactin receptor | **16.32** | **0.92** |
| *SPEF2* | 5:35617946-35814713 | sperm flagellar 2 | 60.51 | * |
| *RNU7-130P* | 5:35633065-35633126 | RNA, U7 small nuclear 130 pseudogene | * | * |
| *IL7R* | 5:35852797-35879705 | interleukin 7 receptor | 53.72 | * |
| *CAPSL* | 5:35904397-35938881 | calcyphosine like | **37.95** | * |
| *UGT3A1* | 5:35951112-36001130 | UDP glycosyltransferase family 3 member A1 | 89.94 | * |
| *UGT3A2* | 5:36035119-36071460 | UDP glycosyltransferase family 3 member A2 | 87.22 | * |
| *LMBRD2* | 5:36098514-36152063 | LMBR1 domain containing 2 | **15.13** | 0.01* |
| *MIR580* | 5:36147994-36148090 | microRNA 580 | * | * |
| *SKP2* | 5:36152091-36184421 | S-phase kinase associated protein 2 | **4.42** | **0.99** |
| *RNU6-1305P* | 5:36155221-36155324 | RNA, U6 small nuclear 1305, pseudogene | * | * |
| *NADK2* | 5:36192694-36242381 | NAD kinase 2, mitochondrial | **13.42** | **0.91** |
| *NADK2-AS1* | 5:36221157-36222004 | NADK2 antisense RNA 1 | * | * |
| *RANBP3L* | 5:36248536-36302216 | RAN binding protein 3 like | 68.51 | * |
| *RNA5SP181* | 5:36485445-36485542 | RNA, 5S ribosomal pseudogene 181 | * | * |
| *SLC1A3* | 5:36606457-36688436 | solute carrier family 1 member 3 | **9.47** | **0.43** |

^#^High ranks (e.g. 0-10%) indicate a gene is more likely to exhibit haploinsufficiency, low ranks (e.g. 90-100%) indicate a gene is more likely to NOT exhibit haploinsufficiency. Huang N, [Lee I](https://europepmc.org/search?query=AUTH:%22Insuk%20Lee%22), [Marcotte EM](https://europepmc.org/authors/0000-0001-8808-180X), [Hurles ME](https://europepmc.org/search?query=AUTH:%22Matthew%20E%20Hurles%22). Characterising and predicting haploinsufficiency in the human genome. Plos Genetics,14 Oct 2010, 6(10):e1001154
DOI:[10.1371/journal.pgen.1001154](https://doi.org/10.1371/journal.pgen.1001154) PMID: 20976243 PMCID: PMC2954820

^&^The pLI score is the probability that a given gene falls into the Haploinsufficient category, and therefore is extremely intolerant of loss-of-function variation. Genes with high pLI scores (pLI ≥ 0.9) are extremely LoF intolerant, whereby genes with low pLI scores (pLI ≤ 0.1) are LoF tolerant. A manuscript describing this dataset and analysis [Karczewski *et al.*, 2020](https://europepmc.org/article/MED/32461654) is available. Further information about the generation of pLI has been published by [Lek *et al.*, 2016](https://europepmc.org/article/MED/27535533).

# .

# - Karczewski KJ^1^ et al.,The mutational constraint spectrum quantified from variation in 141,456 humans. Nature, 27 May 2020, 581(7809):434-443 DOI: [10.1038/s41586-020-2308-7](https://doi.org/10.1038/s41586-020-2308-7) PMID: 32461654. PMCID: PMC7334197

.- Lek M et al. Analysis of protein-coding genetic variation in 60,706 humans. Nature,01 Aug 2016, 536(7616):285-291 DOI: [10.1038/nature19057](https://doi.org/10.1038/nature19057) PMID: 27535533 PMCID: PMC5018207
