# Supplementary material for: Deep Phenotyping and Genetic Characterization of a Cohort of 70 Individuals With 5p Minus Syndrome
Source: Front Genet. 2021 Jul 30;12:645595. doi: 10.3389/fgene.2021.645595 (PMC8362798; doi:10.3389/fgene.2021.645595)
Supplement: Supplementary file 10 [file Presentation_1.PPTX]

## Slide 1
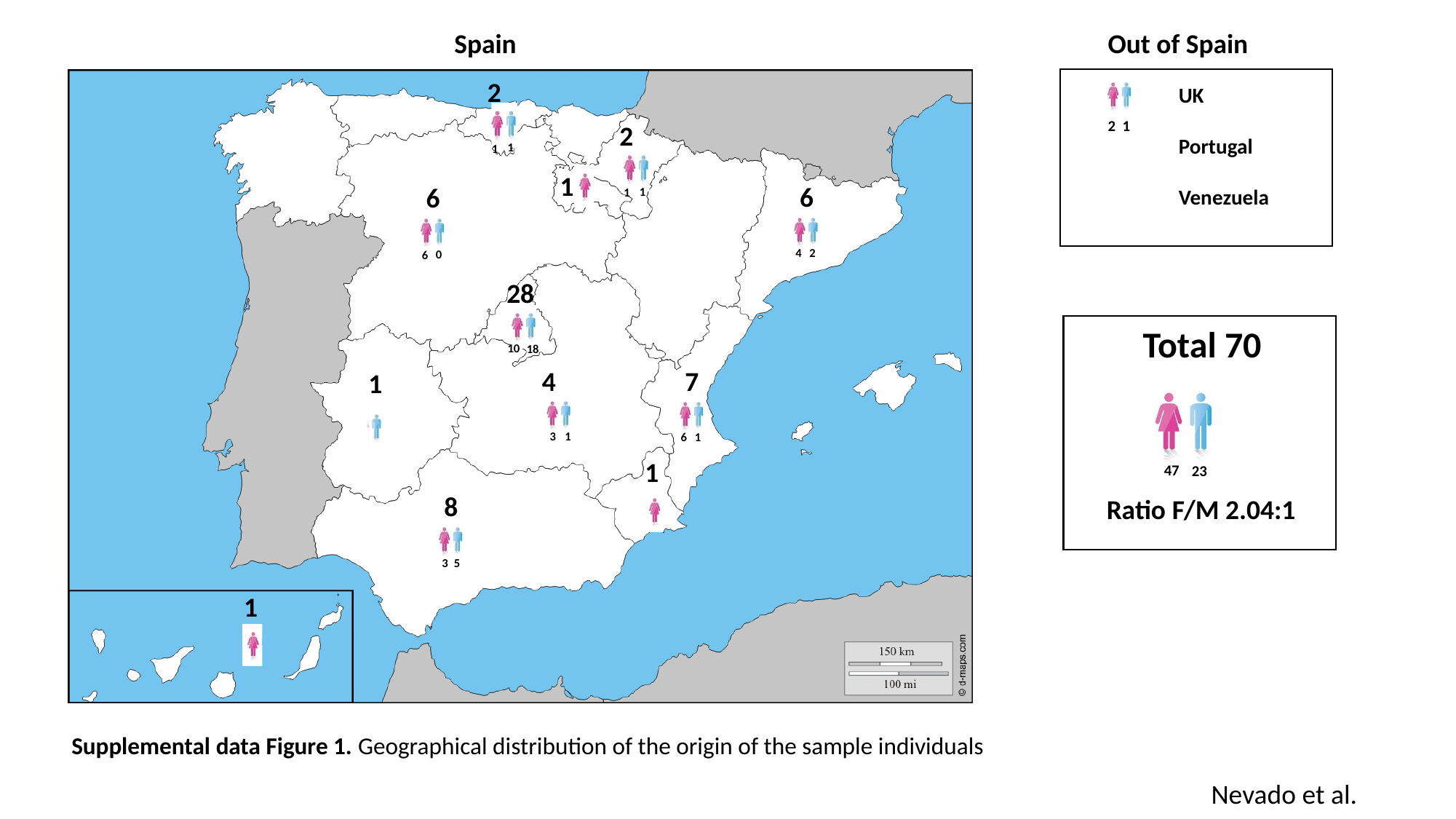

Spain
Out of Spain
2
UK
Portugal
Venezuela
2
1
2
1
1
1
6
6
1
1
4
2
0
6
28
Total 70
10
18
4
7
1
1
3
6
1
1
47
23
8
Ratio F/M 2.04:1
3
5
1
Supplemental data Figure 1. Geographical distribution of the origin of the sample individuals
Nevado et al.
