# Supplementary material for: Deep Phenotyping and Genetic Characterization of a Cohort of 70 Individuals With 5p Minus Syndrome
Source: Front Genet. 2021 Jul 30;12:645595. doi: 10.3389/fgene.2021.645595 (PMC8362798; doi:10.3389/fgene.2021.645595)
Supplement: Supplementary file 11 [file Presentation_2.PPTX]

## Slide 1
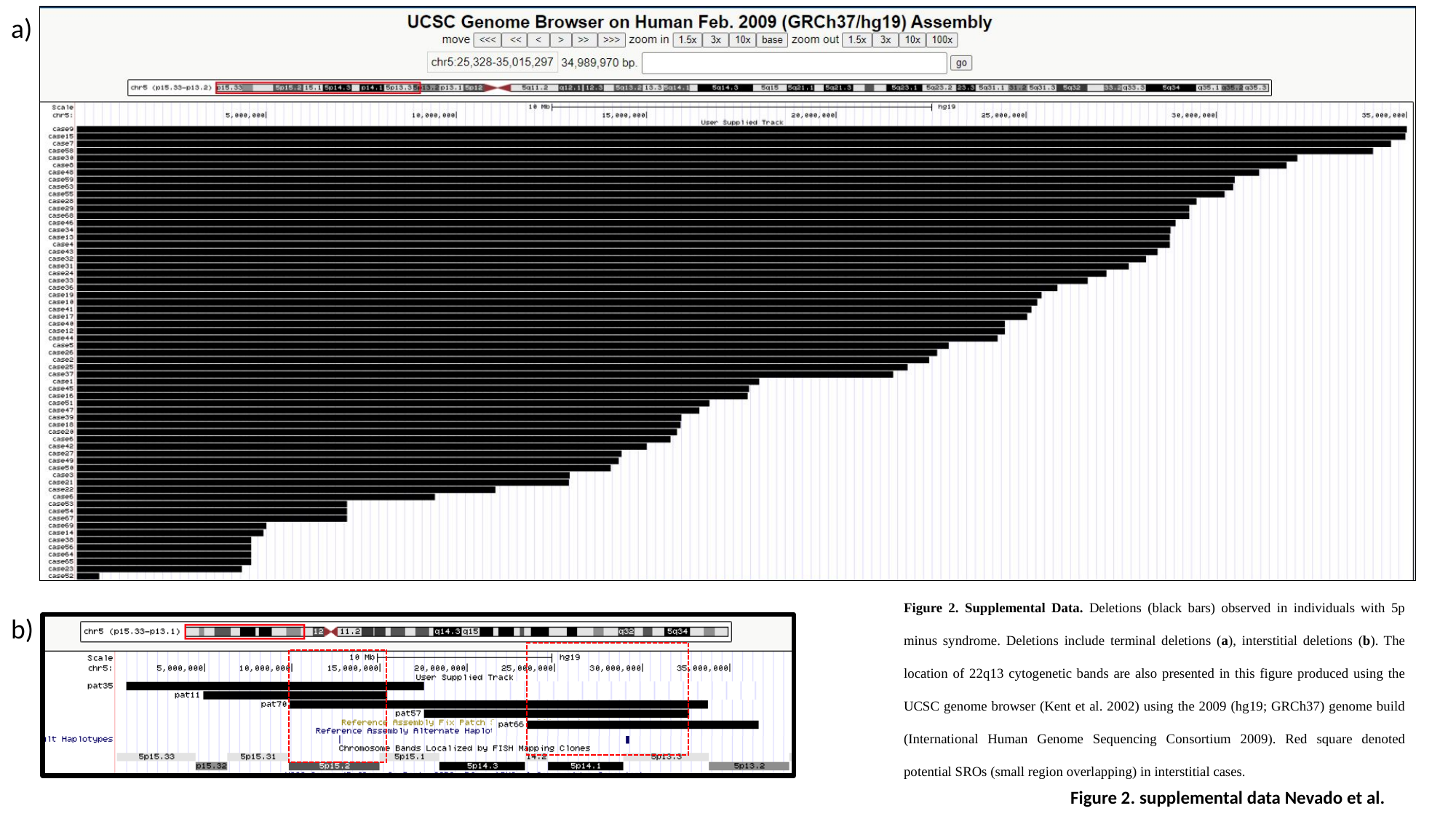

a)
Figure 2. Supplemental Data. Deletions (black bars) observed in individuals with 5p minus syndrome. Deletions include terminal deletions (a), interstitial deletions (b). The location of 22q13 cytogenetic bands are also presented in this figure produced using the UCSC genome browser (Kent et al. 2002) using the 2009 (hg19; GRCh37) genome build (International Human Genome Sequencing Consortium 2009). Red square denoted potential SROs (small region overlapping) in interstitial cases.
b)
Figure 2. supplemental data Nevado et al.
