# Supplementary material for: Deep Phenotyping and Genetic Characterization of a Cohort of 70 Individuals With 5p Minus Syndrome
Source: Front Genet. 2021 Jul 30;12:645595. doi: 10.3389/fgene.2021.645595 (PMC8362798; doi:10.3389/fgene.2021.645595)
Supplement: Supplementary file 12 [file Presentation_3.PPTX]

## Slide 1
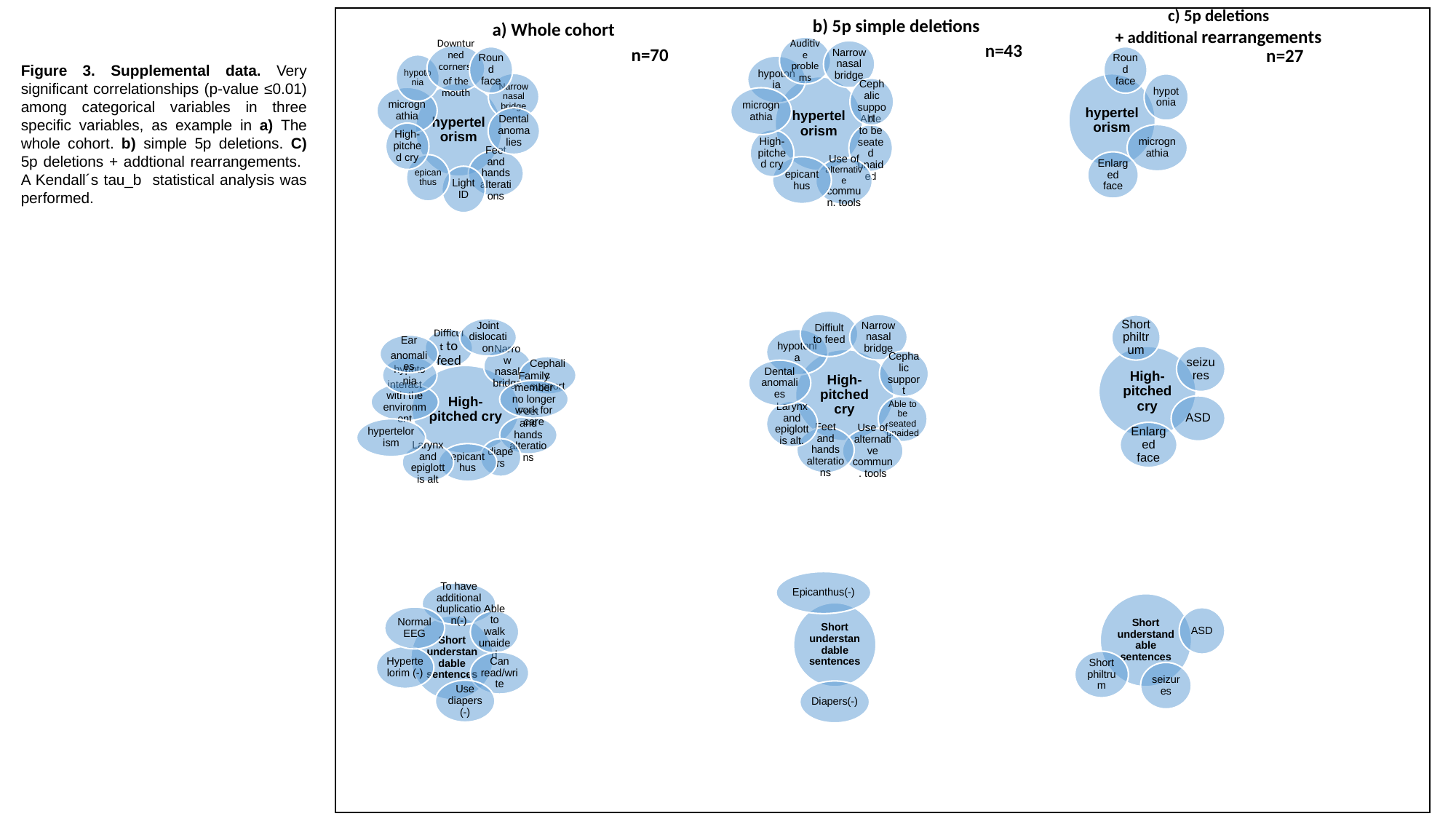

c) 5p deletions
+ additional rearrangements
n=43
n=70
n=27
b) 5p simple deletions
a) Whole cohort
Figure 3. Supplemental data. Very significant correlationships (p-value ≤0.01) among categorical variables in three specific variables, as example in a) The whole cohort. b) simple 5p deletions. C) 5p deletions + addtional rearrangements. A Kendall´s tau_b statistical analysis was performed.
