# Supplementary material for: Deep Phenotyping and Genetic Characterization of a Cohort of 70 Individuals With 5p Minus Syndrome
Source: Front Genet. 2021 Jul 30;12:645595. doi: 10.3389/fgene.2021.645595 (PMC8362798; doi:10.3389/fgene.2021.645595)
Supplement: Supplementary file 13 [file Presentation_4.PPTX]

## Slide 1
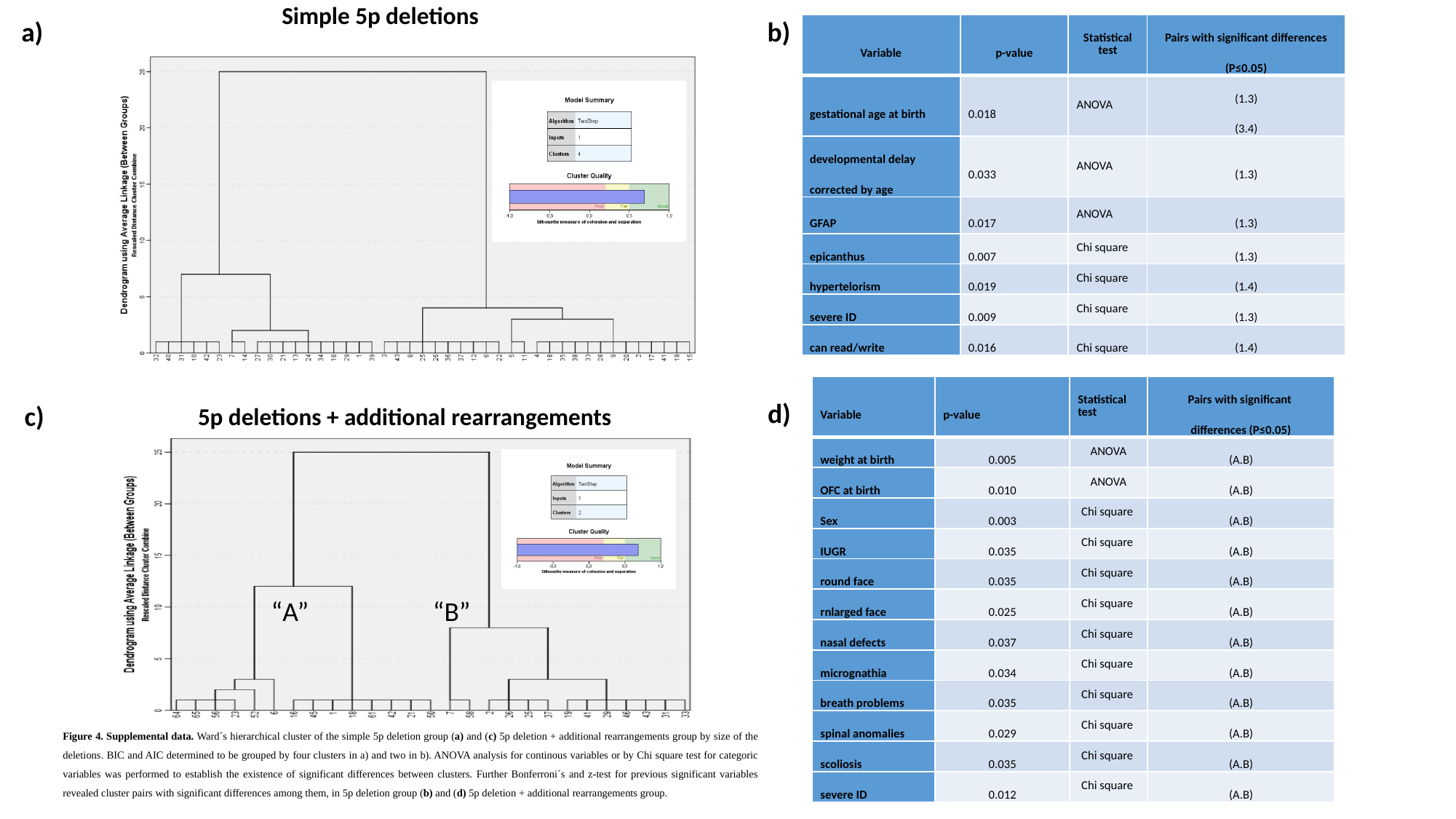

Simple 5p deletions
a)
b)
| Variable | p-value | Statistical test | Pairs with significant differences (P≤0.05) |
| --- | --- | --- | --- |
| gestational age at birth | 0.018 | ANOVA | (1.3) (3.4) |
| developmental delay corrected by age | 0.033 | ANOVA | (1.3) |
| GFAP | 0.017 | ANOVA | (1.3) |
| epicanthus | 0.007 | Chi square | (1.3) |
| hypertelorism | 0.019 | Chi square | (1.4) |
| severe ID | 0.009 | Chi square | (1.3) |
| can read/write | 0.016 | Chi square | (1.4) |
| Variable | p-value | Statistical test | Pairs with significant differences (P≤0.05) |
| --- | --- | --- | --- |
| weight at birth | 0.005 | ANOVA | (A.B) |
| OFC at birth | 0.010 | ANOVA | (A.B) |
| Sex | 0.003 | Chi square | (A.B) |
| IUGR | 0.035 | Chi square | (A.B) |
| round face | 0.035 | Chi square | (A.B) |
| rnlarged face | 0.025 | Chi square | (A.B) |
| nasal defects | 0.037 | Chi square | (A.B) |
| micrognathia | 0.034 | Chi square | (A.B) |
| breath problems | 0.035 | Chi square | (A.B) |
| spinal anomalies | 0.029 | Chi square | (A.B) |
| scoliosis | 0.035 | Chi square | (A.B) |
| severe ID | 0.012 | Chi square | (A.B) |
d)
c)
5p deletions + additional rearrangements
“A”
“B”
Figure 4. Supplemental data. Ward´s hierarchical cluster of the simple 5p deletion group (a) and (c) 5p deletion + additional rearrangements group by size of the deletions. BIC and AIC determined to be grouped by four clusters in a) and two in b). ANOVA analysis for continous variables or by Chi square test for categoric variables was performed to establish the existence of significant differences between clusters. Further Bonferroni´s and z-test for previous significant variables revealed cluster pairs with significant differences among them, in 5p deletion group (b) and (d) 5p deletion + additional rearrangements group.
